# Factorization norms and an inverse theorem for MaxCut

**DOI:** 10.1007/s00208-026-03355-2

**Published:** 2026-02-18

**Authors:** Igor Balla, Lianna Hambardzumyan, István Tomon

**Affiliations:** 1 Faculty of Mathematics and Computer Science, Leipzig University, 04109 Leipzig, Germany; 2 Department of Computer Science, University of Victoria, Victoria, BC V8P 5C2 Canada; 3 Department of Mathematics and Mathematical Statistics, Umeå University, 90736 Umeå, Sweden

**Keywords:** Primary 68Q11, 15A60, Secondary 05C50, 15A18

## Abstract

We prove that Boolean matrices with bounded $\gamma _2$-norm or bounded normalized trace norm must contain a linear-sized all-ones or all-zeros submatrix, verifying a conjecture of Hambardzumyan, Hatami, and Hatami. We also present further structural results about Boolean matrices of bounded $\gamma _2$-norm and discuss applications in communication complexity, operator theory, spectral graph theory, and extremal combinatorics. As a key application, we establish an inverse theorem for MaxCut. A celebrated result of Edwards states that every graph *G* with *m* edges has a cut of size at least $\frac{m}{2}+\frac{\sqrt{8m+1}-1}{8}$, with equality achieved by complete graphs with an odd number of vertices. To contrast this, we prove that if the MaxCut of *G* is at most $\frac{m}{2}+O(\sqrt{m})$, then *G* must contain a clique of size $\Omega (\sqrt{m})$.

## Introduction

For an $m \times n$ matrix *M*, the factorization norm $\gamma _2$ of *M* is defined as

$$\begin{aligned} \gamma _2(M)=\min _{U, V: M=UV}\Vert U\Vert _{{{\,\textrm{row}\,}}}\Vert V\Vert _{{{\,\textrm{col}\,}}}, \end{aligned}$$

where $\Vert U\Vert _{{{\,\textrm{row}\,}}}$ denotes the maximum $\ell _2$-norm of the rows of *U*, and $\Vert V\Vert _{{{\,\textrm{col}\,}}}$ denotes the maximum $\ell _2$-norm of the columns of *V*. This suggests an intuition that the $\gamma _2$-norm can be viewed as a “smooth” version of matrix rank, since the rank is the minimum number of rows of *U* (which equals to the number of columns of *V*) in a factorization. Informally, the factorization norm measures how much the action of a matrix *M* on a vector is distorted when it is factored through the $\ell _2$ space.

Motivated by connections to communication complexity and combinatorics, this paper investigates the structural properties of $\gamma _2$-norm: given the value of $\gamma _2$-norm for a matrix, can we structurally characterize the matrix? Conversely, if the matrix has certain structural properties, how large can its $\gamma _2$-norm be?

Our main result confirms a conjecture of Hambardzumyan, Hatami and Hatami [31] which was motivated by an open question in randomized communication complexity (see Sect. 2.1). They asked whether a Boolean matrix with bounded $\gamma _2$-norm must contain a linear-sized all-ones or all-zeros submatrix [31]. In this work, we answer this question in the affirmative.

### Theorem 1.1

(Main theorem). Let *M* be an $m\times n$ Boolean matrix such that $\gamma _2(M)\le \gamma$. Then *M* contains an $\delta _1 m\times \delta _2 n$ all-zeros or all-ones submatrix such that $\delta _1,\delta _2\ge 2^{-O(\gamma ^3)}$.

More generally, Hambardzumyan, Hatami and Hatami [31] proposed an even stronger version of this conjecture where the condition on the matrices is relaxed to having bounded normalized trace norm. Given an $m\times n$ matrix *M*, the *trace norm* of *M*, denoted by $\Vert M\Vert _{{{\,\textrm{tr}\,}}}$, is the sum of its singular values. Furthermore, the *normalized trace norm* of *M* is defined as $\frac{\Vert M\Vert _{{{\,\textrm{tr}\,}}}}{\sqrt{mn}}$ and it is well known that $\gamma _2(M)$ is an upper bound for this quantity. We verify this conjecture by showing that it is just a simple corollary of Theorem 1.1.

### Corollary 1.2

Let *M* be an $m\times n$ Boolean matrix such that $\frac{\Vert M\Vert _{{{\,\textrm{tr}\,}}}}{\sqrt{mn}}\le \gamma$. Then *M* contains an $\delta _1 m\times \delta _2 n$ all-zeros or all-ones submatrix such that $\delta _1,\delta _2\ge 2^{-O(\gamma ^3)}$.

We highlight another immediate corollary of Theorem 1.1 on arbitrary integer matrices of bounded $\gamma _2$-norm.

### Corollary 1.3

Let *M* be an $m\times n$ integer matrix such that $\gamma _2(M)\le \gamma$. Then *M* contains an $\delta _1 m\times \delta _2 n$ constant submatrix such that $\delta _1,\delta _2\ge 2^{-\gamma ^{O(\gamma )}}$.

Furthermore, we show that Theorem 1.1 is close to optimal, that is, the factor $2^{-O(\gamma ^3)}$ cannot be replaced by anything smaller than $2^{-O(\gamma )}$.

### Theorem 1.4

Let $\gamma \ge 3$ and *n* be sufficiently large with respect to $\gamma$. Then there exists an $n\times n$ Boolean matrix *M* such that $\gamma _2(M)\le \gamma$, and *M* contains no $t\times t$ all-zeros or all-ones submatrix for $t>n2^{-\gamma +3}$.

While the $\gamma _2$-norm of a Boolean matrix can, in general, be difficult to estimate, we show that under a certain structural assumption, it is approximately equal to a simple combinatorial parameter. To this end, we define the *degeneracy* of a Boolean matrix *M* to be the smallest integer *d* such that every submatrix of *M* has a row or a column with at most *d* one entries and we say that a Boolean matrix is *four cycle-free* if it contains no $2\times 2$ all-ones submatrix. In other words, if *M* is the bi-adjacency matrix of a bipartite graph *G*, then the degeneracy of *M* is equal to the degeneracy of *G* and *M* is four cycle-free if and only if *G* has no four cycles. We show that if *M* is four cycle-free, then the $\gamma _2$-norm of *M* is essentially the square-root of its degeneracy.

### Theorem 1.5

Let *M* be a four cycle-free Boolean matrix of degeneracy *d*. Then

$$\begin{aligned} \gamma _2(M)=\Theta \left( \sqrt{d}\right) . \end{aligned}$$

Due to a powerful graph theoretic result of Hunter, Milojević, Sudakov, and Tomon [32], Theorem 1.1 and Theorem 1.5 can be combined to provide the following Zarankiewicz-type result, qualitatively generalizing both.

### Theorem 1.6

For every $\gamma >0$ there exists a constant $C >0$ such that the following holds. Let *M* be an $n\times n$ Boolean matrix such that $\gamma _2(M)\le \gamma$. If *M* has at least *Ctn* one-entries, then *M* contains a $t\times t$ all-ones submatrix.

The factorization norm $\gamma _2$ and, more broadly, factorization theory for Banach spaces have been a central and influential topic in functional analysis, originating with the work of Kwapień [40] and Maurey [48], as well as being implicitly present in an earlier work by Grothendieck [27]. Moreover, the $\gamma _2$-norm has also found far-reaching applications across theoretical computer science. These include lower bounds on quantum and randomized communication protocols in communication complexity [46], bounds on hereditary discrepancy in discrepancy theory [50], the algorithmic version of the celebrated Bourgain–Tzafriri theorem on the column subset selection problem [57], a connection to margin complexity in learning theory [45], and several applications in differential privacy [17, 21, 49]. Thus, understanding the fundamental properties of the $\gamma _2$-norm is strongly motivated by applications in these areas.

### The MaxCut problem

Given a graph *G*, a *cut* in *G* is a partition of the vertices into two parts, together with all the edges having exactly one vertex in each part. The size of the cut is the number of its edges, and the *MaxCut* of *G* is the maximum size of a cut. Algorithmic and theoretical properties of the MaxCut are extensively studied [1, 7, 14, 15, 18, 30].

If *G* has *m* edges, then *G* has a cut of size at least *m*/2, as this is the expected size of the cut resulting from a uniform random partition of the vertex set. A fundamental result of Edwards [14, 15] states that this simple bound can be improved to $m/2+(\sqrt{8m+1}-1)/8$, which is sharp in case *G* is a complete graph on an odd number of vertices. In general, this shows that every graph with *m* edges has MaxCut of size at least $m/2+\Omega (\sqrt{m})$. On the other hand, all known examples of graphs with MaxCut of size $m/2+O(\sqrt{m})$ are close to complete graphs or the disjoint union of complete graphs. Motivated by this, we establish a strong structural property of graphs with MaxCut of size $m/2+O(\sqrt{m})$: they contain a complete subgraph of size $\Omega (\sqrt{m})$.

#### Theorem 1.7

Let $\alpha >0$ and let *G* be a graph with *m* edges containing no cut of size larger than $m/2+\alpha \sqrt{m}$. Then *G* contains a clique of size $2^{-O(\alpha ^9)}\sqrt{m}$.

This result connects to a line of research initiated by Erdős and Lovász in the 1970 s (see [18]), who studied MaxCut under forbidden subgraph conditions. Specifically, they considered the maximum cut size in graphs that avoid a fixed subgraph *H*, a problem which received substantial interest [1, 2, 7, 25]. Alon, Krivelevich, and Sudakov [1] showed that for every graph *H* there exists a constant $\varepsilon _H>0$ such that every *H*-free graph with *m* edges has a cut of size at least $m/2+\Omega _H(m^{1/2+\varepsilon _H})$. They further conjectured that a stronger bound $m/2+\Omega _H(m^{3/4+\varepsilon _H'})$ holds for some appropriate $\varepsilon _H'>0$. In order to prove this, it is clearly enough to consider the case when *H* is a complete graph. A closely related result of Räty, Sudakov, and Tomon [52] shows that regular graphs with edge density between $1/2+\varepsilon$ and $1-\varepsilon$ have a cut of size $m/2+\Omega _{\varepsilon }(m^{5/8})$.

Our Theorem 1.7 addresses the extreme case of the Alon, Krivelevich, Sudakov conjecture, where *H* is a clique whose size is comparable to the host graph, specifically $H=K_{\Omega (\sqrt{m})}$.

At first glance, Theorem 1.7 may appear unrelated to factorization norms. However, somewhat surprisingly, our proof of this theorem relies on Theorem 1.1 and Corollary 1.2. Specifically, we show that if *A* is the adjacency matrix of *G*, then *G* has a cut of size $m/2+\Omega (\Vert A\Vert _{{{\,\textrm{tr}\,}}})$, improving a recent result of [53]. From this, we get an upper bound on the trace norm of *A*, enabling a direct application of Corollary 1.2. The proof of Theorem 1.7 is presented in Sect. 9.

**Organization.** In Sect. 2, we present applications and motivations of our results in communication complexity, operator theory, combinatorics and discrepancy theory. We prove Theorem 1.1, Corollary 1.2 and Corollary 1.3 in Sect. 5 after some preliminary results in Sect. 4. Then, we prove Theorem 1.4 in Sect. 6, Theorem 1.5 and Theorem 1.6 in Sect. 7, and Theorem 1.7 in Sect. 9.

## Applications

### Communication complexity

The $\gamma _2$-norm was introduced into communication complexity by the seminal paper of Linial and Shraibman [46]. The $\gamma _2$-norm of the matrix *M* and its approximate version lower bound the following basic and well-studied communication models:

deterministic ${{\,\textrm{D}\,}}(M)$, deterministic with oracle access to the Equality function ${{\,\textrm{D}\,}}^{{{\,\textrm{EQ}\,}}}(M)$, public-coin randomized communication ${{\,\textrm{R}\,}}(M)$, and quantum communication with shared entanglement ${{\,\textrm{Q}\,}}^{*}(M)$. More precisely, for a Boolean matrix *M*, we have

$$\begin{aligned} \log \gamma _2(M) \le {{\,\textrm{D}\,}}^{{{\,\textrm{EQ}\,}}}(M) \le {{\,\textrm{D}\,}}(M), \end{aligned}$$

where the first inequality is proven in [31] and the weaker inequality of $\log \gamma _2(M) \le {{\,\textrm{D}\,}}(M)$ was initially proven in [46].

We note that the first inequality implies that Theorem 1.5 strengthens [35, Theorem 1.5], which establishes that a four cycle-free Boolean matrix has bounded ${{\,\textrm{D}\,}}^{{{\,\textrm{EQ}\,}}}$ complexity if and only if it has bounded degeneracy. In contrast, Theorem 1.5 shows that this equivalence holds under the weaker assumption of bounded $\gamma _2$-norm.

For the $n \times n$ matrix *M*, let the *approximate* $\gamma _2$*-norm* of *M*, denoted by $\tilde{\gamma }_2(M)$, be the minimum $\gamma _2$-norm of an $n \times n$ matrix $M'$ that satisfies $|M(i,j)-M'(i,j)|\le \frac{1}{3}$ for every entry $(i,j)\in [n]\times [n]$. Then, the following inequality is proved in [46],

1 $$\begin{aligned} \log \tilde{\gamma }_2(M) \lesssim {{\,\textrm{Q}\,}}^{*}(M)\le {{\,\textrm{R}\,}}(M) \le O\left( \tilde{\gamma }_2(M)\right) ^2. \end{aligned}$$

#### Structure and randomized communication

All-ones and all-zeros submatrices of a Boolean matrix play a central role in communication complexity, since they serve as the fundamental building blocks of communication protocols. Finding such submatrices of large size often leads to efficient protocol design via recursion techniques (e.g., [51]), while their absence can lead to strong lower bounds against communication protocols in various models. For instance, a classical result states that if the deterministic communication complexity of a Boolean matrix is low, then the matrix can be partitioned into large all-ones and all-zeros submatrices. This structural insight has been instrumental in obtaining strong lower bounds in the deterministic model.

In contrast, the randomized model of communication is much less understood from this structural viewpoint: no such decomposition exists in general for randomized communication complexity—as illustrated, for instance, by the identity matrix. Developing a structural characterization of randomized complexity would shed light on the broader question of understanding the power of randomness in communication (how randomness enhances the power of communication protocols). As a step in this direction, Refs. [31, 34] independently initiated the study of constant-cost randomized communication complexity as an extreme case of randomness in communication, a topic that has since attracted significant attention [19, 20, 35]. Even though a full partitioning into large all-ones and all-zeros submatrices is impossible in the randomized setting, one might still hope to find such large submatrices in Boolean matrices of constant-cost randomized communication. This intuition is captured by the following conjecture, made independently by a number of groups.

##### Conjecture 2.1

([13, 26, 31]). Every Boolean matrix with randomized communication complexity bounded by a constant contains a linear-sized all-zeros or all-ones submatrix.

In view of the lower bound in (1), one natural strategy to settle the conjecture would be to show that if the matrix has bounded approximate $\gamma _2$-norm, then it has a linear-sized all-zeros or all-ones submatrix. However, until now, this question has remained open even for the exact $\gamma _2$-norm itself. As such, this problem has stood as a major barrier to progress on Conjecture 2.1. Motivated by this obstacle, Hambardzumyan, Hatami, and Hatami [31, Conjecture ii] conjectured that such structure exists not only for matrices with bounded $\gamma _2$-norm but even for the ones that satisfy the relaxed condition of having a bounded normalized trace norm. This is indeed a relaxation, as $\frac{\Vert M\Vert _{{{\,\textrm{tr}\,}}}}{\sqrt{mn}} \le \gamma _2(M)$. In [31], this conjecture regarding the normalized trace norm was proven for a special class of matrices known as group lifts, for which it can be deduced from a quantitative version of Cohen’s idempotent theorem.

In this work, we resolve the conjecture of [31] regarding matrices with bounded normalized trace norm in full generality. Our proof combines linear algebraic, analytic and combinatorial techniques. Specifically, Corollary 1.2 shows that every Boolean matrix with bounded normalized trace norm contains a linear-sized all-zeros or all-ones submatrix. This result eliminates a major bottleneck toward Conjecture 2.1 and offers strong evidence in its favor.

#### Separation between the $\gamma _2$-norm and randomized communication.

Linial and Shraibman [46] proposed the problem of whether $\tilde{\gamma }_2(M)$ in (1) can be replaced with $\gamma _2(M)$ to get a stronger lower bound for ${{\,\textrm{R}\,}}(M)$. However, this was recently disproved by Cheung, Hatami, Hosseini, and Shirley [11] in a strong sense, who constructed an $n\times n$ Boolean matrix *M* such that $\gamma _2(M)\ge \Omega (n^{1/32})$ and ${{\,\textrm{R}\,}}(M)=O(\log \log n)$. Their main technical result is as follows.

Let $1\le q\le p$ be integers, and let $P=P(q,p)$ be the $qp\times qp$ Boolean matrix, whose rows and columns are indexed by the elements of $[q]\times [p]$, and its entries are given by $P[(x,x'),(y,y')]=1$ iff $xy+x'=y'$. Furthermore, let $P_p=P_p(q,p)$ be the matrix defined almost identically, but $P[(x,x'),(y,y')]=1$ iff $xy+x'=y'$ holds modulo *p*. In [11], it is proved, by technical applications of Fourier analysis, that $\gamma _2(P_p)=\Omega (q^{1/8})$ if $q\le \sqrt{p}$, and $\gamma _2(P)=\Omega (q^{1/8})$ if $q\le p^{1/3}$.

However, note that *P* and $P_p$ are the incidence matrices of points and lines, so they are four cycle-free. Therefore, Theorem 1.5 immediately implies the following improvements.

##### Theorem 2.2

Let $1\le q\le p-1$. Then $\gamma _2(P_p)=\Theta (\sqrt{q})$ and $\gamma _2(P)=\Theta (\min \{\sqrt{q},p^{1/4}\})$.

##### Proof

Given $x,x',y$, there is a unique $y'$ such that $xy+x'=y' \pmod {p}$, and also given $x,y,y'$, there is a a unique $x'$ such that $xy+x'=y' \pmod {p}$. Therefore, each row and column of $P_p$ contains *q* one entries, so the degeneracy of $P_p$ is also *q*. By Theorem 1.5, we get $\gamma _2(P_p)=\Theta (\sqrt{q})$.

Now let us consider *P*, and let us only prove the lower bound, we leave the upper bound as an exercise. We may assume that $q\le \sqrt{p}$, as otherwise $P(\sqrt{p},p)$ is a submatrix of *P*(*q*, *p*) and we use that the $\gamma _2$-norm of a submatrix is always at most the $\gamma _2$-norm of the matrix. Given $x\in [q]$, there are at least *qp*/4 solutions of $xy+x'=y'$ with $y\in [q]$ and $x',y'\in [p]$. Therefore, the number of one entries of *P* is at least $pq^2/4$, which means that the degeneracy of *P* is at least *q*/4. Hence, by Theorem 1.5, $\gamma _2(P_p)=\Omega (\sqrt{q})$. $\square$

Very recently, a similar result was obtained by Cheung, Hatami, Hosseini, Nikolov, Pitassi, and Shirley [10], based on similar ideas. One of their main technical lemmas shows that if *M* is a four-cycle free Boolean matrix, then $\gamma _2(M)\ge ||M||_F^2/\sqrt{2\Delta }$, where $\Delta$ is the maximum degree of the associated bipartite graph. This gives the same bound as Theorem 1.5 in case the bipartite graph is close to regular and otherwise, Theorem 1.5 is stronger.

#### The Log-rank conjecture

The celebrated Log-rank conjecture of Lovász and Saks [44] proposes that the deterministic communication complexity of a rank *r* matrix is bounded by $(\log r)^{O(1)}$. This conjecture is equivalent to the statement that every $m\times n$ Boolean matrix *M* of rank *r* contains an all-ones or all-zeros submatrix of size $m2^{-(\log r)^{O(1)}}\times n2^{-(\log r)^{O(1)}}$. This conjecture is still wide open, where the best known lower bound on the size of the all-ones or all-zeros submatrix is $m2^{-O(\sqrt{r})}\times n2^{-O(\sqrt{r})}$, due to a recent result of Sudakov and Tomon [55], slightly improving an earlier result of Lovett [42].

The Log-rank conjecture is closely related to Theorem 1.1. Indeed, as observed by Linial and Shraibman [46], if *M* is a Boolean matrix of rank *r*, then $\gamma _2(M)\le \sqrt{r}$. While our main result in Theorem 1.1 yields a submatrix whose size is smaller than the best known bounds on the Log-rank conjecture, it is applicable to a much larger class of matrices. On the other hand, Theorem 1.4 shows that one cannot hope to settle the Log-rank conjecture—or even improve the current best bound—by obtaining a larger than $m2^{-O(\sqrt{r})}\times n2^{-O(\sqrt{r})}$ all-ones or all-zeros submatrix solely by tightening the dependence on $\gamma _2$ in Theorem 1.1.

### Operator theory and harmonic analysis

In operator theory, the question of characterizing the idempotent Schur multipliers in terms of contractive idempotents is widely open. In [31], using the fact that the operator norm induced by a Schur multiplier *M* coincides with its $\gamma _2$-norm, it was shown that this question is equivalent to the structural characterization of matrices with bounded $\gamma _2$-norm. In particular, it is equivalent to the following conjecture. A *blocky matrix* is a blow-up of a permutation matrix. Formally, *B* is a blocky matrix if there is partition $X_0,\dots ,X_m$ of its rows and a partition $Y_0,\dots ,Y_m$ of its columns such that $B[X_i\times Y_i]$ is the all-ones matrix for $i\in [m]$, and $B[X_i\times Y_j]=0$ for $i \ne j$.

#### Conjecture 2.3

([31]) Let *M* be an $m\times n$ Boolean matrix such that $\gamma _2(M)\le \gamma$. Then there exists $c_{\gamma }$, depending only on $\gamma$, such that *M* is a $\pm 1$-linear combination of at most $c_{\gamma }$ blocky-matrices.

It is not difficult to see that Conjecture 2.3, if true, implies Theorem 1.1 (see [31, Lemma 3.5]). On the other hand, in an upcoming work, Goh and Hatami [24] builds on  Theorem 1.1 to prove the following substantial evidence towards this conjecture. They show that if *M* is a Boolean matrix with |*M*| one-entries such that $\gamma _2(M)\le \gamma$, then there is a blocky-matrix *B* such that $|B|\ge |M|/2^{2^{O(\gamma )}}$, and *B* is “contained” in *M*, i.e. $M_{i,j}\ge B_{i,j}$ for every entry (*i*, *j*). In another, even more recent work, Goh and Hatami [23] prove that Conjecture 2.3 holds up to a polylogarithmic error term. More precisely, *M* can be written as a signed sum of at most $2^{O(\gamma ^7)}(\log \min \{m,n\})^2$ blocky-matrices.

From the perspective of harmonic analysis,  Conjecture 2.3 is the analogue of Cohen’s celebrated idempotent theorem for the algebra of Schur multipliers. Specifically, Cohen’s theorem—made quantitative for finite Abelian groups by Green-Sanders [28, 29]—states that if $f: G \rightarrow \{0,1\}$ is a function on a finite Abelian group *G* with bounded Fourier $\ell _1$-norm, $\Vert \hat{f}\Vert _1 \le \ell$, then *f* can be written as the $\pm 1$-linear combination of $c_{\ell }$ many indicator functions of cosets of *G*, that is $f = \sum _{i}^{c_{\ell }} \pm 1_{H_{i} + a_i}$. Equivalently, every idempotent of the Fourier algebra of *G* can be expressed as a linear combination of many contractive idempotents $c_{\ell }$—precisely the type of structural result one seeks in the algebra of Schur multipliers. For more details on this connection, see [31].

Another connection to Cohen’s idempotent theorem is for a special class of matrices known as group lifts, that is, matrices *M* that can be written as $M(x,y) = f(y - x)$ for some function $f: G \rightarrow \mathbb {R}$ over a finite abelian group *G*. Under such lift, we know that $\Vert \hat{f}\Vert _1 = \gamma _2(M)$, and an indicator function of a coset corresponds to a blow-up of an identity matrix. Hence, Conjecture 2.3 holds for group lifts as a direct consequence of Cohen’s theorem.

Given this parallel between the $\gamma _2$-norm and the Fourier $\ell _1$-norm, Theorem 1.1 can be view as an analogue of a theorem of Shpilka, Tal, and Volk [56], which shows that if a Boolean function *f* has $\Vert \hat{f}\Vert _1 \le \ell$, then there is an affine subspace $V \subseteq \{0,1\}^n$ of co-dimension at most $\ell ^2$ such that *f* is constant on *V*. Theorem 1.1 extends this result from Boolean functions to Boolean matrices by proving the existence of a large constant submatrix.

### Spectral graph theory

Graphs, whose adjacency matrices have bounded $\gamma _2$-norm naturally appear in spectral graph theory, and in the study of equiangular lines.

#### Graphs of bounded smallest eigenvalue

A central topic of spectral graph theory is to understand the structure of graphs having smallest eigenvalue at least $-\lambda$, see e.g. Koolen, Cao and Yang [38] for a survey. It is easy to show that if *G* is a nonempty graph, then the smallest eigenvalue of its adjacency matrix is at most $-1$, with equality if and only if *G* is the disjoint union of cliques. A celebrated theorem of Cameron, Goethels, Seidel, and Shult [8] from 1972 settles the case $\lambda = 2$ and establishes a connection to root systems, and more recently, Koolen, Yang and Yang [41] obtained a partial characterization in the case $\lambda = 3$.

It turns out that if a graph has smallest eigenvalue at least $-\lambda$, then the $\gamma _2$-norm of its adjacency matrix is at most $2\lambda$, see Lemma 8.2. Hence, our results immediately apply to such graphs. Moreover, we establish the following strengthening of Theorem 1.6, which shows that graphs of bounded smallest eigenvalue contain cliques of size comparable to their average degree. This result also follows from a structure theorem of Kim, Koolen and Yang [39]. However, while their proof uses Ramsey theoretic arguments, our proof relies on Theorem 1.6 instead.

##### Theorem 2.4

Let $\lambda >0$ and let *G* be a graph with average degree *d* and smallest eigenvalue at least $-\lambda$. Then *G* contains a clique of size $\Omega _{\lambda }(d)$.

We present the proof of Theorem 2.4 in Sect. 8.

#### Equiangular lines

A collection of lines $\mathcal {L}$ in $\mathbb {R}^d$ through the origin is *equiangular* with angle $\alpha$ if the angle between any two lines of $\mathcal {L}$ is $\alpha$. It is an old problem of Lemmens and Siedel [43] to determine the maximum size of an equiangular set of lines with angle $\alpha$ in $\mathbb {R}^d$. This problem was essentially solved recently by Jiang, Tidor, Yao, Zhang and Zhao [37], building on the work of Balla, Dräxler, Keevash, and Sudakov [6]. For the extensive history of equiangular lines and further quantitative improvements on this problem, we refer the interested reader to [4].

A collection of equiangular lines with angle $\alpha$ can be represented by a symmetric Boolean matrix (or equivalently a graph) as follows. Pick a unit direction vector $v_{\ell }$ for each line $\ell$, and let *M* be the matrix whose rows and columns are represented by the elements of $\mathcal {L}$, and set $M(\ell _1,\ell _2)=1$ if the angle between $v_{\ell _1}$ and $v_{\ell _2}$ is $\alpha$, and set $M(\ell _1,\ell _2)=0$ if this angle is $\pi -\alpha$. Set the diagonal entries 0. If $t=\cos (\alpha )$, then $M=\frac{1}{2t}N-\frac{1}{2t}I+J$, where *N* is the Gram matrix of the vectors $\{v_{\ell }\}_{\ell \in \mathcal {L}}$. From this, $\gamma _2(N)\le 1/t+1$. Hence, if $\alpha$ is bounded away from $\pi /2$, the Boolean matrix *M* has bounded $\gamma _2$-norm, so our results immediately apply, giving interesting results about the Ramsey properties of the graphs associated with equiangular lines. In particular, we get the following immediate corollary of Theorem 1.1.

##### Theorem 2.5

Let $\delta >0$, then there exists $c=c(\delta )>0$ such that the following holds. Let $\alpha \in [0,\pi /2-\delta )$, and let $\mathcal {A}$ and $\mathcal {B}$ be two sets of lines through the origin in a real space such that the angle between $\ell _1$ and $\ell _2$ is $\alpha$ for every $(\ell _1,\ell _2)\in \mathcal {A}\times \mathcal {B}$. Then, given any choice of direction vectors $v_{\ell }$ for every $\ell \in \mathcal {A}\cup \mathcal {B}$, there exist $\mathcal {A}'\subset \mathcal {A}$ and $\mathcal {B}'\subset \mathcal {B}$ such that $|\mathcal {A}'|\ge c|\mathcal {A}|$, $|\mathcal {B}'|\ge c|\mathcal {B}|$, and either, the angle between $v_{\ell _1}$ and $v_{\ell _2}$ is $\alpha$ for every $(\ell _1,\ell _2)\in \mathcal {A}'\times \mathcal {B}'$, or it is $\pi -\alpha$ for every $(\ell _1,\ell _2)\in \mathcal {A}'\times \mathcal {B}'$.

We highlight that the study of Ramsey properties of graphs associated to equiangular lines played a crucial role in the resolution of the above described problem of Lemmens and Siedel, see [6, 37] for further details.

### Discrepancy theory

The $\gamma _2$-norm has important applications in discrepancy theory as well. Let *M* be an $m\times n$ matrix, then the *combinatorial discrepancy* of *M* is defined as

$$\begin{aligned} {{\,\textrm{DISC}\,}}(M)=\min _{x\in \{-1,1\}^n} ||Mx||_{\infty }. \end{aligned}$$

Here, $\Vert \cdot \Vert _{\infty }$ is the maximum absolute value of the entries. (To avoid confusion, later in this paper, we will introduce another type of discrepancy, which we denote by ${{\,\textrm{disc}\,}}(.)$.) Moreover, the *hereditary discrepancy* of *M* is defined as ${{\,\textrm{HERDISC}\,}}(M)=\max _{N\subset M} {{\,\textrm{DISC}\,}}(N),$ where the maximum is taken over all submatrices *N* of *M*. If $\mathcal {F}$ is set system on a ground set *X*, then ${{\,\textrm{DISC}\,}}(\mathcal {F})={{\,\textrm{DISC}\,}}(M)$ and ${{\,\textrm{HERDISC}\,}}(\mathcal {F})={{\,\textrm{HERDISC}\,}}(M)$, where *M* is the incidence matrix of $\mathcal {F}$, with rows representing the sets. In combinatorial terms, the discrepancy of $\mathcal {F}$ is the minimal *k* for which there is a red-blue coloring of the elements of *X* such that the numbers of red and blue elements in each set of $\mathcal {F}$ differ by at most *k*.

Combinatorial discrepancy theory has its roots in the study of irregularities of distributions and it has become a highly active area of research since the 80’s [5]. It has also found profound applications in computer science, see the book of Chazelle [9] for a general reference. The following general inequality of Matoušek, Nikolov, and Talwar [50] establishes a sharp relation between the $\gamma _2$-norm and the hereditary discrepancy of arbitrary matrices:

$$\begin{aligned} \Omega \left( \frac{\gamma _2(M)}{\log m}\right) \le {{\,\textrm{HERDISC}\,}}(M)=O\left( \gamma _2(M)\sqrt{\log m}\right) . \end{aligned}$$

Combining this theorem with Theorem 1.5, we immediately get that if *M* is a four cycle-free Boolean matrix of degeneracy *d*, then ${{\,\textrm{HERDISC}\,}}(M)$ and $\sqrt{d}$ are equal up to logarithmic factors. For example, if *G* is the incidence graph of *n* points and *m* lines in the plane, then *G* is four cycle-free and the Szemerédi–Trotter theorem implies that it has degeneracy $O(n^{1/3})$. This bound is also the best possible, so we get close to optimal bounds on the discrepancy of geometric set systems generated by lines, recovering the results of Chazelle and Lvov [12].

## Preliminaries

In this paper, we use mostly standard linear algebraic and graph theoretic notation.

### Graph theory

Given a graph *G*, $e(G)=|E(G)|$ denotes the number of edges of *G*. If $U\subset V(G)$, then *G*[*U*] is the subgraph of *G* induced on the vertex set *U*. We make use of the following fundamental result of extremal graph theory.

#### Theorem 3.1

(Turán’s theorem [58]) Let *G* be a graph on *n* vertices such that the complement of *G* has average degree at most *d*. Then *G* contains a clique of size at least $\frac{n}{d+1}.$ Equivalently, if the complement has *t* edges, then there is a clique of size at least $\frac{n^2}{2t+n}.$

### Basics of linear algebra

Given a real vector *v*, ||*v*|| denotes the $\ell _2$-norm of *v*. The *Hadamard product* (or entry-wise product) of two matrices *A* and *B* of size $m\times n$ is the $m\times n$ sized matrix $A\circ B$ defined as $(A\circ B)_{i,j}=A_{i,j}B_{i,j}$. We make use of the well known *Cauchy interlacing theorem*, which we state here for the reader’s convenience.

#### Lemma 3.2

Let *M* be an $n\times n$ real symmetric matrix with eigenvalues $\lambda _1\ge \dots \ge \lambda _n$, and let *N* be an $m\times m$ principal submatrix of *M* with eigenvalues $\mu _1\ge \dots \ge \mu _m$. Then $\lambda _i\ge \mu _i\ge \lambda _{i+n-m}$ for $i=1,\dots ,m$.

### Matrix norms

Given two matrices $A,B\in \mathbb {R}^{m\times n}$, their Frobenius inner product is defined as

$$\begin{aligned} \langle A,B\rangle =\sum _{i=1}^m\sum _{j=1}^n A_{i,j}B_{i,j}={{\,\textrm{tr}\,}}\left( AB^T\right) . \end{aligned}$$

Let *M* be an $m\times n$ matrix with singular values $\sigma _1,\dots ,\sigma _k$, $k=\min \{m,n\}$. The *Frobenius norm* of *M* can be defined in multiple equivalent ways:

$$\begin{aligned} ||M||_F^2:=\langle M,M\rangle ={{\,\textrm{tr}\,}}\left( MM^T\right) =\sum _{i=1}^{k}\sigma _i^2. \end{aligned}$$

The *trace-norm* of *M* is

$$\begin{aligned} ||M||_{{{\,\textrm{tr}\,}}}=\sigma _1+\dots +\sigma _k. \end{aligned}$$

Finally, we write

$$\begin{aligned} |M|=\sum _{i,j}\left| M_{i,j}\right| , \end{aligned}$$

which, in case *M* is a Boolean matrix, is just the number of one entries.

### The $\gamma _2$-norm

The $\gamma _2$-norm of a matrix *M* is defined as

$$\begin{aligned} \gamma _2(M)=\min _{M=UV}||U||_{{{\,\textrm{row}\,}}}||V||_{{{\,\textrm{col}\,}}}, \end{aligned}$$

where $||U||_{{{\,\textrm{row}\,}}}=||U||_{2\rightarrow \infty }$ denotes the maximum $\ell _2$-norm of the rows of *U*, and $||V||_{{{\,\textrm{col}\,}}}=||V||_{1\rightarrow 2}$ denotes the maximum $\ell _2$-norm of the columns of *V*. Here, we collect some basic properties of the $\gamma _2$-norm, see e.g. [47] as a general reference.

Let $M\in \mathbb {R}^{m\times n}$ and let *N* be a real matrix.

1. If $$c\in \mathbb {R}$$, then $$\gamma _2(cM)=|c|\gamma _2(M)$$.
2. (monotonicity) If *N* is a submatrix of *M*, then $\gamma _2(N)\le \gamma _2(M)$.
3. (subadditivity) If *M* and *N* have the same size, then $\gamma _2(M+N)\le \gamma _2(M)+\gamma _2(N)$.
4. $\gamma _2(M)=\max ||M\circ (u v^T)||_{{{\,\textrm{tr}\,}}},$ where the maximum is over all unit vectors $u\in \mathbb {R}^m$, $v\in \mathbb {R}^{n}$.
5. $$\gamma _2(M)\ge \frac{1}{\sqrt{mn}}||M||_{{{\,\textrm{tr}\,}}}.$$
6. Duplicating rows or columns of *M* does not change the $\gamma _2$-norm.
7. $$\gamma _2(M)\le ||M||_{{{\,\textrm{row}\,}}}$$ and $$\gamma _2(M)\le ||M||_{{{\,\textrm{col}\,}}}$$.
8. If *M* is a non-zero Boolean matrix, then $\gamma _2(M)\ge 1$, with equality if and only if *M* is a blocky matrix.
9. If *M* is a Boolean matrix, then $\gamma _2(M)\le \sqrt{\text{ rank }(M)}$.
10. $\gamma _2(M\otimes N)=\gamma _2(M)\gamma _2(N)$, where $\otimes$ denotes the tensor product.
11. If *M* and *N* have the same size, then $\gamma _2(M\circ N)\le \gamma _2(M)\gamma _2(N)$

Here, we remark that (11) follows from (10) as $M\circ N$ is a submatrix of $M\otimes N$.

## Sparsifying matrices

In this section, we prove a technical result showing that if *M* is a Boolean matrix with bounded $\gamma _2$-norm such that the density of zero entries is $\varepsilon >0$, then one can boost this to density at least $1-\varepsilon$ by passing to a linear-sized submatrix of *M*. Such a result can be proved in at least two different ways. One approach is based on noting that Boolean matrices of bounded $\gamma _2$-norm have bounded VC-dimension, and then invoke the *ultra-strong regularity lemma* for such matrices, see e.g. [22]. Another approach is based on the discrepancy method, recently used in connection to the Log-rank conjecture [33, 42, 55]. We present the latter approach, as it provides quantitatively better bounds. We mostly follow the proof of Lemma 4.2 in [33].

Given an $m\times n$ Boolean matrix *M*, let $p(M)=|M|/mn$, that is, *p*(*M*) is the density of one entries. Define the *discrepancy* of *M* as

$$\begin{aligned} {{\,\textrm{disc}\,}}(M)=\max _{A\subset [m], B\subset [n]} \big ||M[A\times B]|-p(M)|A||B|\big |. \end{aligned}$$

### Lemma 4.1

Let *M* be a Boolean matrix such that $p(M)<1-\varepsilon$ and $\gamma _2(M)\le \gamma$. Then

$$\begin{aligned} {{\,\textrm{disc}\,}}(M)=\Omega (\varepsilon |M|/\gamma ). \end{aligned}$$

### Proof

Let $m\times n$ be the size of *M*, and let $N=M-p(M)J$. Then

$$\begin{aligned} {{\,\textrm{disc}\,}}(M)=\max _{A\subset [m], B\subset [n]} |N[A\times B]|, \end{aligned}$$

where the right-hand-side is just the *cut-norm* of *N*. The dual of the $\gamma _2$-norm is defined as

$$\begin{aligned} \gamma _2^*(N)=\max \left| \sum _{i=1}^{m}\sum _{j=1}^n N_{i,j}\left\langle x_i,y_j\right\rangle \right| , \end{aligned}$$

where the maximum is taken over all vectors $x_1, \dots , x_m, y_1, \dots , y_n\in \mathbb {R}^{d}$ and all dimensions *d* such that $\Vert x_i\Vert , \Vert y_j\Vert \le 1$. It follows from Grothendieck’s inequality that the $\gamma _2^*$-norm and cut-norm are equal up to absolute constant factors (see e.g. [46]), so we have

$$\begin{aligned} {{\,\textrm{disc}\,}}(M)=\Omega \left( \gamma _2^*(N)\right) . \end{aligned}$$

We have $\gamma _2(N)\le \gamma _2(p(M)J)+\gamma _2(M)\le 1+\gamma$, so there exists a factorization $N=UV$ such that $||U||_{{{\,\textrm{row}\,}}}\le 1+\gamma$ and $||V||_{{{\,\textrm{col}\,}}}\le 1$. For $i=1,\dots ,m$, let $x_i=u_i/(1+\gamma )$, where $u_1,\dots ,u_m$ are the rows of *U*, and let $y_1,\dots ,y_n$ be the columns of *V*. Then $||x_i||,||y_j||\le 1$, so

$$\begin{aligned} \gamma _2^{*}(N)&\ge \left| \sum _{i=1}^{m}\sum _{j=1}^n N_{i,j}\left\langle x_i,y_j\right\rangle \right| =\frac{1}{\gamma +1}\sum _{i=1}^m\sum _{j=1}^n N_{i,j}^2=\frac{1}{\gamma +1}\sum _{i=1}^m\sum _{j=1}^n \left( M_{i,j}-p(M)\right) ^2\\&=\frac{1}{\gamma +1}\left( |M|(1-p(M))^2+(mn-|M|)p(M)^2\right) \ge \frac{\varepsilon |M|}{4(\gamma +1)}. \end{aligned}$$

The last inequality holds as if $p(M)\le 1/2$, then the first term in the brackets is at least |*M*|/4, and if $p(M)\ge 1/2$, then the second term is at least $\varepsilon mn/4\ge \varepsilon |M|/4$. This finishes the proof. $\square$

We also use the following consequence of Claim 2.2 from [55].

### Lemma 4.2

Let *M* be an $m\times n$ Boolean matrix. Then *M* contains an $m/2\times n/2$ submatrix *N* such that $p(N)\le p(M)-\Omega \left( \frac{{{\,\textrm{disc}\,}}(M)}{mn}\right)$.

Combining the previous two lemmas, we deduce the following.

### Lemma 4.3

Let *M* be an $m\times n$ Boolean matrix such that $\gamma _2(M)\le \gamma$, and $p(M)\le 1-\varepsilon$. Then *M* contains an $m/2\times n/2$ submatrix *N* such that $p(N)\le p(M)(1-\Omega (\varepsilon /\gamma ))$.

### Proof

By Lemma 4.2, *M* contains an $m/2\times n/2$ submatrix *N* such that $p(N)\le p(M)-\Omega (\frac{{{\,\textrm{disc}\,}}(M)}{mn})$. However, by Lemma 4.1, we have ${{\,\textrm{disc}\,}}(M)=\Omega (\varepsilon |M|/\gamma )=\Omega (\varepsilon p(M)mn/\gamma )$, finishing the proof. $\square$

### Lemma 4.4

Let *M* be an $m\times n$ Boolean matrix such that $\gamma _2(M)\le \gamma$, and $p(M)\le 1-\varepsilon$, and let $0<\delta <1/2$. Then *M* contains an $m'\times n'$ submatrix *N* such that every row of *N* has at most $\delta n'$ one entries, every column has at most $\delta m'$ one entries, and $m'/m, n'/n > 2^{-O((\gamma /\varepsilon )\log (1/\delta ))}$.

### Proof

Define a sequence of submatrices $M_0,M_1,\dots$ of *M* as follows. Let $M_0=M$. If $M_i$ of size $m_i\times n_i$ is already defined such that $p_i=p(M_i)$, let $M_{i+1}$ be the $m_{i}/2\times n_{i}/2$ sized submatrix of $M_i$ given by  Lemma 4.3, so that $p(M_{i+1})\le p(M_i)(1-\Omega (\varepsilon /\gamma ))$. Then $m_i=m2^{-i}$, $n_i=n2^{-i}$, and

$$\begin{aligned} p\left( M_i\right) \le p(M)\left( 1-\Omega (\varepsilon /\gamma )\right) ^i\le p(M)e^{-\Omega \left( i\varepsilon /\gamma \right) }. \end{aligned}$$

Hence, if $I = \lceil c\frac{\gamma }{\varepsilon } \log (1/\delta ) \rceil$ for some *c* sufficiently large, we have $p(M_I)\le \delta /4$. Delete all rows of $M_I$ which contain more that $\delta n_I/2$ one entries, and delete all columns of $M_I$ which contain more than $\delta m_I/2$ one entries. Then we deleted at most $m_I/2$ rows and $n_I/2$ columns. If we let *N* be the resulting matrix, then *N* has size $m'\times n'$ with $m'\ge m_I/2= 2^{-I-1}m$ and $n'\ge n_I/2=2^{-I-1}n$, and each row of *N* contains at most $\delta m_I/2 \le \delta m'$ one entries, and each column contains at most $\delta n_I/2 \le \delta n'$ one entries. $\square$

## Large all-ones or all-zeros submatrices

In this section, we prove Theorem 1.1 and Corollary 1.2. First, we show that Theorem 1.1 indeed implies Corollary 1.2. To this end, we prove the following simple lemma.

### Lemma 5.1

Let *M* be an $m\times n$ matrix such that $||M||_{{{\,\textrm{tr}\,}}}\le \gamma \sqrt{mn}$. Then for every $\varepsilon \in (0,1/2]$, *M* contains an $m'\times n'$ submatrix $M'$ such that $m'\ge (1-\varepsilon )m$, $n'\ge (1-\varepsilon )n$, and $\gamma _2(M')\le \gamma /\varepsilon$.

### Proof

Let $k=\min \{m,n\}$, and let $\sigma _1,\dots ,\sigma _k$ be the singular values of *M*. Let $M=A\Sigma B$ be the singular value decomposition of *M*, that is, *A* is an $m\times k$ matrix, whose columns are orthonormal vectors, *B* is $k\times n$ matrix, whose rows are orthonormal vectors, and $\Sigma$ is a $k\times k$ diagonal matrix, whose diagonal entries are $\sigma _1,\dots ,\sigma _{k}$. Let $U=A\Sigma ^{1/2}$ and $V=\Sigma ^{1/2} B$, so $M=UV$. Observe that

$$\begin{aligned} ||U||_F^2=||V||_F^2=\sum _{i=1}^k\sigma _i=||M||_{{{\,\textrm{tr}\,}}}\le \gamma \sqrt{mn}. \end{aligned}$$

Let $U'$ be the submatrix of *U* we get by keeping the $m'=(1-\varepsilon )m$ rows with smallest $\ell _2$-norms, and let $V'$ be the submatrix of *V* we get by keeping the $n'=(1-\varepsilon ) n$ columns with the smallest $\ell _2$-norms. Then $||U'||_{{{\,\textrm{row}\,}}}^2\le (\gamma /\varepsilon )\sqrt{n/m}$ and $||V'||_{{{\,\textrm{col}\,}}}^2\le (\gamma /\varepsilon )\sqrt{m/n}$. But then $M'=U'V'$ is an $m'\times n'$ submatrix of *M* such that $\gamma _2(M')\le ||U'||_{{{\,\textrm{row}\,}}}||V'||_{{{\,\textrm{col}\,}}}\le \gamma /\varepsilon$. $\square$

### Proof of Corollary 1.2 assuming Theorem 1.1

By applying Lemma 5.1 with $\varepsilon =1/2$ to *M*, we get an $m/2\times n/2$ submatrix $M'$ such that $\gamma _2\left( M'\right) \le 2\gamma$. But then by Theorem 1.1, $M'$ contains a $t\times u$ all-zeros or all-ones submatrix for some $\frac{t}{m},\frac{u}{m}\ge 2^{-O(\gamma ^3)}$, finishing the proof. $\square$

We now turn towards the proof of our main result, Theorem 1.1. To this end, we first argue that it is enough to consider the case $m=n$. Indeed, let *M* be an $m\times n$ matrix such that $\gamma _2(M)\le \gamma$. Let $M^{\otimes }$ be the matrix we get by repeating every row of *M* exactly *n* times and then repeating every column of the resulting matrix *m* times, i.e. $M^{\otimes } = M \otimes J$ where *J* is the $n \times m$ all-ones matrix. Then $M^{\otimes }$ is an $n'\times n'$ matrix with $n'=mn$ and $\gamma _2(M^{\otimes })=\gamma _2(M)\le \gamma$. Assume that $M^{\otimes }$ contains a $t'\times t'$ all-zeros or all-ones submatrix, where $t'\ge cn'$ for some $c=c(\gamma )>0$. Then removing repeated rows and columns, this gives a $t\times u$ sized all-zeros or all-ones submatrix of *M* with $t=\lceil \frac{t'}{n}\rceil$ and $u=\lceil \frac{t'}{m}\rceil$, so that $\frac{t}{m},\frac{u}{n}\ge c$.

Therefore, in the rest of the argument, we assume that $m=n$. Instead of Theorem 1.1, we prove the slightly stronger result that if at least half of the entries are zero, then one can find a large all-zero submatrix.

### Theorem 5.2

Let *M* be an $n\times n$ Boolean matrix such that $\gamma _2(M)\le \gamma$ and $p(M)\le 1/2$. Then *M* contains a $t\times t$ all-zeros submatrix for some $t=n2^{-O(\gamma ^3)}$.

Note that Theorem 1.1 follows from this immediately: if $|M|\le n^2/2$, we apply Theorem 5.2 to find a large all-zero submatrix. However, if $|M|\ge n^2/2$, we apply Theorem 5.2 to the matrix $J-M$. By noting that $\gamma _2(J-M)\le \gamma +1$, this guarantees a large all-ones submatrix in *M*. The key technical result underpinning the proof of Theorem 5.2 is the following lemma, which tells us that we can find a large submatrix of *M* with significantly smaller $\gamma _2$-norm. Our main theorem then follows by repeated application of this lemma.

### Lemma 5.3

Let *M* be an $n\times n$ non-zero Boolean matrix such that $\gamma _2(M)\le \gamma$, and $p(M)\le 1/2$. Then *M* contains an $m\times m$ submatrix *N* such that $\gamma _2(N)\le \gamma -\Omega (1/\gamma )$, $m\ge n2^{-O(\gamma )}$, and $p(N)\le 1/2$.

### Proof of Theorem 5.2 assuming Lemma 5.3

Define a sequence of submatrices of *M* as follows. Let $M_0=M$. If $M_i$ of size $n_i\times n_i$ is already defined such that $p(M_i)\le 1/2$ and $M_i$ is non-zero, then let $M_{i+1}$ be the $n_{i+1}\times n_{i+1}$ submatrix of $M_i$ given by Lemma 5.3, so that $\gamma _2(M_{i+1})<\gamma _2(M_{i+1})- c / \gamma _2(M_{i+1})$ for some constant $c > 0$, $p(M_{i+1})\le 1/2$ and $n _{i+1}=n_i2^{-O(\gamma )}$. By induction, we have $\gamma (M_{i})\le \gamma - c i /\gamma$ and $n_{i}=n2^{-O(\gamma i)}$. On the other hand, we trivially have $\gamma _2(M_i) \ge 0$ and so if $i > \gamma ^2 / c$, then $M_i$ must not be defined. Since $M_{i+1}$ is only not defined in case $M_{i}$ is the all-zero matrix, if we let *i* be the smallest integer such that $M_i$ is the all-zero matrix, then we have established that $i \le \lceil \gamma ^2 / c \rceil$ and so $M_i$ is the desired $n_i \times n_i$ matrix with $n_i = n 2^{-O(i \gamma )} = n2^{-O(\gamma ^3)}$. $\square$

The rest of this section is devoted to the proof of Lemma 5.3. The following definition and accompanying result are crucial.

### Definition 1

Let *M* be an $m\times n$ Boolean matrix with factorization $M=UV$, and let $u_1,\dots ,u_m$ be the row vectors of *U*, and $v_1,\dots ,v_n$ be the column vectors of *V*. For $r\in [m]$, say that row *r* is *brilliant* (with respect to (*M*, *U*, *V*)) if $u_r\ne 0$, and denoting by $d_r$ the number of 1 entries in the *r*-th row of *M*, we have

$$\begin{aligned} \sum _{i=1}^{m}\left\langle u_r,u_i\right\rangle ^2\ge d_r. \end{aligned}$$

Similarly, for $c\in [n]$, say that column *c* is *brilliant* (with respect to (*M*, *U*, *V*)) if $v_c\ne 0$, and denoting by $d'_c$ the number of 1 entries in the *c*-th column of *M*, we have

$$\begin{aligned} \sum _{i=1}^{n}\left\langle v_c,v_i\right\rangle ^2\ge d'_c. \end{aligned}$$

### Lemma 5.4

Let *M* be a non-zero Boolean matrix with factorization $M=UV$. Then there exists either a brilliant row or a brilliant column.

### Proof

Assume that none of the rows or columns are brilliant. We make use of the following identity: if *x*, *y* are real vectors of the same dimension, then $\langle xx^T,yy^T\rangle =\langle x,y\rangle ^2$.

Let $u_1,\dots ,u_m$ be the rows of *U*, and $v_1,\dots ,v_n$ be the columns of *V*. We have

2 $$\begin{aligned} 0\le \left| \left| \sum _{i=1}^m u_iu_i^T-\sum _{j=1}^n v_jv_j^T\right| \right| _F^2=\sum _{r=1}^{m}\sum _{i=1}^m \left\langle u_r,u_i\right\rangle ^2+\sum _{c=1}^{n}\sum _{j=1}^n \left\langle v_c,v_j\right\rangle ^2-2\sum _{i=1}^{m}\sum _{j=1}^n\left\langle u_i,v_j\right\rangle ^2. \end{aligned}$$

Here, $\sum _{i=1}^{m}\sum _{j=1}^n\langle u_i,v_j\rangle ^2=|M|$ is the number of one entries of *M*. If none of the rows are brilliant, then

$$\begin{aligned} \sum _{r=1}^{m}\sum _{i=1}^m \left\langle u_r,u_i\right\rangle ^2<\sum _{r=1}^m d_r=|M|, \end{aligned}$$

and similarly, if none of the columns are brilliant, then

$$\begin{aligned} \sum _{c=1}^{n}\sum _{j=1}^n \left\langle v_c,v_j\right\rangle ^2<\sum _{c=1}^n d'_c=|M|. \end{aligned}$$

This contradicts (2), so we must have either a brilliant row or a brilliant column. $\square$

### Proof of Lemma 5.3

Let $M_0$ be an $n_0\times n_0$ submatrix of *M* such that $n_0=n2^{-O(\gamma )}$, and each row and each column of $M_0$ has at most $0.1n_0$ one entries. Applying Lemma 4.4 with $\varepsilon =1/2$ and $\delta =0.1$, we can find such a submatrix. Write $M_0=U_0V_0$, where $||U_0||_{{{\,\textrm{row}\,}}}^2\le \gamma$ and $||V_0||_{{{\,\textrm{col}\,}}}^2\le \gamma$.

Repeat the following procedure. If $M_i=U_iV_i$ is already defined, where the size of $M_i$ is $m_i\times n_i$, proceed as follows. Stop if either (a) $M_i$ is an all-zero matrix, or (b) $m_i<n_0/2$, or (c) $n_i<n_0/2$. Otherwise, proceed depending on the following two cases.

**Case 1.** There exists a brilliant row *r* with respect to $(M_i,U_i,V_i)$.

Let $u_1,\dots ,u_{m_i}$ be the rows of $U_i$. Let $u_j'$ be the projection of $u_j$ to the orthogonal complement of $u_r$, that is,

$$\begin{aligned} u_j'=u_j-\frac{\left\langle u_r,u_j\right\rangle }{\left| \left| u_r\right| \right| ^2}u_r. \end{aligned}$$

Let $U_{i+1}$ be the matrix, whose rows are $u_1',\dots ,u_{m_i}'$. Furthermore, let $V_{i+1}$ be the submatrix of $V_i$, in which we keep those columns *v* of $V_i$ that satisfy $\langle u_r,v\rangle =0$. In other words, we keep the *j*-th column of $V_i$ if $(M_i)_{r,j}=0$. Finally, set $M_{i+1}=U_{i+1}V_{i+1}$. We observe that $M_{i+1}$ is a submatrix of $M_{i}$. This follows from the fact that if $\langle u_r,v\rangle =0$, then $\langle u_j',v\rangle =\langle u_j,v\rangle$ for all $j\in [m_i]$. Moreover, the size of $M_{i+1}$ is $m_i\times (n_{i}-t_i)$, where $t_i$ is the number of one entries in row *r* of $M_i$. Furthermore,

$$\begin{aligned} \left| \left| u_j'\right| \right| ^2=\left| \left| u_j-\frac{\left\langle u_r,u_j\right\rangle }{\left| \left| u_r\right| \right| ^2}u_r\right| \right| ^2=\left| \left| u_j\right| \right| ^2-\frac{\left\langle u_r,u_j\right\rangle ^2}{\left| \left| u_r\right| \right| ^2}\le \left| \left| u_j\right| \right| ^2-\frac{\left\langle u_r,u_j\right\rangle ^2}{\gamma }, \end{aligned}$$

from which

$$\begin{aligned} \left| \left| U_{i+1}\right| \right| _F^2=\sum _{j=1}^{m_i}\left| \left| u_j'\right| \right| ^2\le \sum _{j=1}^{m_i}\left| \left| u_j\right| \right| ^2-\frac{1}{\gamma }\sum _{j=1}^{m_i}\left\langle u_r,u_j\right\rangle ^2\le \left| \left| U_{i}\right| \right| _F^2-\frac{t_i}{\gamma }, \end{aligned}$$

where the last inequality follows by our assumption that row *r* is brilliant.

**Case 2.** There exists no brilliant row with respect to $(M_i,U_i,V_i)$.

Then by Lemma 5.4, there exists a brilliant column *c*. We proceed similarly as in the previous case, but with the roles of *U* and *V* swapped. Let $v_1,\dots ,v_{n_i}$ be the columns of $V_i$, and let $v_j'$ be the projection of $v_j$ to the orthogonal complement of $v_c$. Let $V_{i+1}$ be the matrix, whose columns are $v_1',\dots ,v_{n_i}'$. Furthermore, let $U_{i+1}$ be the submatrix of $U_i$, in which we keep those rows *u* of $U_i$ such that $\langle u,v_c\rangle =0$. Finally, set $M_{i+1}=U_{i+1}V_{i+1}$. We observe again that $M_{i+1}$ is a submatrix of $M_{i}$, and the size of $M_{i+1}$ is $(m_i-t_i)\times n_{i}$, where $t_i$ is the number of one entries in column *c*. Finally,

$$\begin{aligned} \left| \left| V_{i+1}\right| \right| _F^2\le \left| \left| V_{i}\right| \right| _F^2-\frac{t_i}{\gamma }. \end{aligned}$$

We make some observations about our process.

1. If at step *i* we are in Case 1, then we get $M_{i+1}$ from $M_i$ by removing columns, and if we are in Case 2, we only remove rows.
2. If for any *i* we invoked Case 2, that is, there is no brilliant row at some step *i*, then there are no brilliant rows at any later step either. Indeed, in Case 2, we are only removing rows, so a non-brilliant row cannot become brilliant.
3. The process ends in a finite number of steps. Indeed, at each step, we turn a non-zero vector of $U_i$ or $V_i$ into a zero vector by projection.

Let $i=I$ be the index for which we stopped, and recall that this means that either (a) $M_I$ is the all-zero matrix, or (b) $n_I<n_0/2$ or (c) $m_I<n_0/2$. Note that at each step *i*, the number of rows or columns decreases by some *t* (possibly $t=0$), where *t* is upper bounded by the number of one entries in a row or a column of $M_0$, in particular $t\le 0.1n_0$. Hence, we must have that $m_I>n_0/4$ and $n_I>n_0/4$. If (a) $M_I$ is the all-zero matrix, we are done by taking *N* to be any $n_0/4\times n_0/4$ submatrix of $M_I$, so assume that either (b) or (c) happened.

First, assume that (b) happened, so $n_I<n_0/2$. By (1) and (2), this is only possible if at every step, we invoked Case 1. Therefore, we have $m_I=n_0$, and $n_I=n_0-\sum _{i=0}^{I-1}t_i$, which implies $\sum _{i=0}^{I-1}t_i\ge n_0/2$. On the other hand, we have

$$\begin{aligned} \left| \left| U_{I}\right| \right| _F^2\le ||U_0||_F^2-\sum _{i=0}^{I-1}\frac{t_i}{\gamma }<||U_0||_F^2-\frac{n_0}{2\gamma }\le n_0\gamma -\frac{n_0}{2\gamma }. \end{aligned}$$

Let $U'$ be the submatrix of $U_I$ in which we keep those rows *u* that satisfy $||u||^2\le \gamma -\frac{1}{4\gamma }$, and let $m'$ be the number of rows of $U'$. Then

$$\begin{aligned} n_0\left( \gamma -\frac{1}{2\gamma }\right) \ge ||U_I||_F^2\ge (n_0-m')\left( \gamma -\frac{1}{4\gamma }\right) , \end{aligned}$$

from which we conclude that $m'\ge n_0/(4\gamma ^2)$. Set $N'=U'V_I$, then $N'$ is an $m'\times n_I$ submatrix of *M* such that

$$\begin{aligned} \gamma _2(N')\le \left| \left| U'\right| \right| _{{{\,\textrm{row}\,}}}||V_I||_{{{\,\textrm{col}\,}}}\le \sqrt{\gamma -\frac{1}{4\gamma }}\cdot \sqrt{\gamma }=\gamma -\Omega \left( 1/\gamma \right) , \end{aligned}$$

and $m',n_I=\Omega _{\gamma }(n)$. As $n_I\ge n_0/4$, each row of $N'$ has at most $0.1n_0\le n_I/2$ one entries. Hence, $p(N')\le 1/2$. Set $m=\min \{m',n_I\}\ge n_0/(4\gamma ^2)=n2^{-O(\gamma )}$, then by a simple averaging argument, we can find an $m\times m$ submatrix *N* of $N'$ such that $p(N)\le 1/2$. This *N* suffices.

Finally, we consider the case if (c) happened, that is, if $m_I<n_0/2$. In this case we proceed very similarly as in the previous case, so we only give a brief outline of the argument. Let *J* be the first index for which we invoked Case 2 in the process. Using (2), it follows that we invoked Case 2 for every index $i>J$ as well. We have $n_I=n_J\ge n_0/2$ and $m_I=n_0-\sum _{i=J}^{I-1}t_i$. As before, we conclude that

$$\begin{aligned} ||V_{I}||_F^2\le ||V_J||_F^2-\sum _{i=J}^{I-1}\frac{t_i}{\gamma }<||V_J||_F^2-\frac{n_0}{2\gamma }\le n_J\gamma -\frac{n_J}{2\gamma }. \end{aligned}$$

From this, we deduce that if $V'$ is the submatrix of $V_I$ formed by those columns *v* that satisfy $||v||^2\le \gamma -\frac{1}{4\gamma }$, then $V_J$ has at least $n_J/(4\gamma ^2)\ge n_0/(8\gamma ^2)$ columns. Setting $N'=U_IV'$, the rest of the proof proceeds in the same manner as in case (b). $\square$

Finally, we prove Corollary 1.3.

### Proof of Corollary 1.3

Observe that if *M* is an integer matrix such that $\gamma _2(M)\le \gamma$, then all entries of *M* are in the set $S=\{-\lfloor \gamma \rfloor ,\dots ,\lfloor \gamma \rfloor \}$. Hence, there exists some integer $t\in S$ such that at least $mn/(2\gamma +1)$ entries of *M* are equal to *t*. Let $q\in \mathbb {R}[X]$ be the polynomial defined as $q(x)=1+c\prod _{k\in S\setminus \{t\}}(x-k)$, where $c\ne 0$ is chosen such that $q(t)=0$. Then $q(k)=1$ for every $k\in S\setminus \{t\}$, and $q(t)=0$. Let *N* be the $m\times n$ Boolean matrix defined as $N_{i,j}=q(M_{i,j})$. Then writing $q(x)=\sum _{i=0}^{|S|-1}a_ix^i$, we have $N=\sum _{i=0}^{|S|-1}a_i M^{\circ i}$, where $M^{\circ i}$ denotes the Hadamard product $M\circ \dots \circ M$ with *i* terms. Hence, by property (11) of the $\gamma _2$-norm, we have

$$\begin{aligned} \gamma ':=\gamma _2(N)\le \sum _{i=0}^{|S|-1}|a_i|\gamma ^i=\gamma ^{O(\gamma )}. \end{aligned}$$

Here, $p(N)\le 1-1/(2\gamma +1)$, so we can apply Lemma 4.4 to find an $m'\times n'$ submatrix $N'$ such that $p(N')\le 1/2$ and $\frac{m'}{m},\frac{n'}{n}\ge 2^{-O(\gamma ^2 \gamma ')}=2^{-\gamma ^{O(\gamma )}}$. Finally, applying Lemma 5.3, we get that $N'$ contains an all-zero submatrix $N''$ of size $m''\times n''$ with $\frac{m''}{m'},\frac{n''}{n'}\ge 2^{-O(\gamma '^3)}=2^{-\gamma ^{O(\gamma )}}$. But then $N''$ corresponds to a submatrix of *M* of size $m2^{-\gamma ^{O(\gamma )}}\times n2^{-\gamma ^{O(\gamma )}}$ in which every entry is *t*, finishing the proof. $\square$

## Construction of tight example

In this section, we present a construction of matrices with bounded $\gamma _2$-norm and no large all-ones and all-zeros submatrices.

### Proof of Theorem 1.4

Let $\ell =\lfloor \gamma \rfloor \ge 3$, and let *k* be an integer sufficiently large with respect to $\ell$. Let *S* be a *k* element ground set, let $p=k^{3/2-\ell }$, and let $\mathcal {F}$ be a random sample of the $\ell$-element subsets of *S*, where each $\ell$-element set is included independently with probability *p*. If we let $X=|\mathcal {F}|$, then $$\mathbb {E}(X)=p\left( {\begin{array}{c}k\\ \ell \end{array}}\right) =\Omega _{\ell }(k^{3/2})$$ and by standard concentration arguments, $\mathbb {P}(X>\mathbb {E}(X)/2)>0.9$. Let *Y* be the number of pairs of sets in $\mathcal {F}$ whose intersection has size at least two. Then $\mathbb {E}(Y)<p^2k^{2\ell -2}=k$, so by Markov’s inequality, $\mathbb {P}(Y<10k)\ge 0.9$. Furthermore, let $Y'$ be the number of pairs of sets in $\mathcal {F}$ whose intersection has size exactly 1. Then $\mathbb {E}(Y')\le p^2 k^{2\ell -1}=k^2$, so by Markov’s inequality, $\mathbb {P}(Y'<10k^2)\ge 0.9$. Finally, let $T\subset S$ be any set of size *k*/2 and let $Z_T$ be the number of elements of $\mathcal {F}$ completely contained in *T*. Then $$\mathbb {E}(Z_T)=p\left( {\begin{array}{c}k/2\\ \ell \end{array}}\right) <2^{-\ell }\mathbb {E}(X)$$. By the multiplicative Chernoff inequality, we can write

$$\begin{aligned} \mathbb {P}\left( Z_T\ge 2\mathbb {E}\left( Z_T\right) \right) \le \exp \left( -\frac{1}{3}\mathbb {E}\left( Z_T\right) \right) \le \exp \left( -\Omega _{\ell }\left( k^{3/2}\right) \right) . \end{aligned}$$

Hence, as the number of *k*/2 element subsets of *S* is at most $2^k$, a simple application of the union bound implies

$$\begin{aligned} \mathbb {P}(\forall T\subset S, |T|=k/2: Z_T\le 2\mathbb {E}(Z_T))>0.9. \end{aligned}$$

In conclusion, there exists a choice for $\mathcal {F}$ such that $X>\mathbb {E}(X)/2$, $Y<10k$, $Y'<10k^2$, and $Z_T\le 2\mathbb {E}(Z_T)\le 2\cdot 2^{-\ell }\mathbb {E}(X)$ for every *k*/2 element set *T*. For each pair of sets intersecting in more than one element in $\mathcal {F}$, remove one of them from $\mathcal {F}$ and let $\mathcal {F}'$ be the resulting set. If we let $n=|\mathcal {F}'|/2=\Omega _{\ell }(k^{3/2})$, then $n>X/2-5k\ge \mathbb {E}(X)/4-5k =\Omega _{\ell }(k^{3/2})$, and thus $Z_T\le 8\cdot 2^{-\ell }n$ for every *T*.

Define the $n\times n$ matrix *M* as follows. Let $\mathcal {A}\cup \mathcal {B}$ be an arbitrary partition of $\mathcal {F}'$ into two *n* element sets. Let *U* be the $n\times k$ matrix whose rows are the characteristic vectors of the elements of $\mathcal {A}$, let *V* be the $k\times n$ matrix whose columns are the characteristic vectors of the elements of $\mathcal {B}$, and set $M=UV$. As each row of *U* and each column of *V* is a zero–one vector with $\ell$ one entries, we have $\gamma _2(M)\le ||U||_{{{\,\textrm{row}\,}}}||V||_{{{\,\textrm{col}\,}}}=\ell$. Also, *M* is a Boolean matrix, which is guaranteed by the fact that any two distinct sets in $\mathcal {F}'$ intersect in 0 or 1 elements. The number of one entries of *M* is at most $Y'<10k^2=o(n^2)$, which shows that *M* contains no $n2^{-\gamma }\times n2^{-\gamma }$ sized all-ones submatrix if *n* is sufficiently large. Finally, *M* contains no $t\times t$ all-zeros submatrix if $t>8\cdot 2^{-\ell }n$. Indeed, a $t\times t$ all-zeros submatrix corresponds to subfamilies $\mathcal {A}'\subset \mathcal {A}$ and $\mathcal {B}'\subset \mathcal {B}$ of sizes *t* such that every element of $\mathcal {A}'$ is disjoint from every element of $\mathcal {B}'$. In other words, if $T_1=\bigcup _{A\in \mathcal {A}'}A$ and $T_2=\bigcup _{B\in \mathcal {B}'} B$, then $T_1$ and $T_2$ are disjoint. But then at least one of $T_1$ or $T_2$ has size at most *k*/2, without loss of generality, $|T_1|\le k/2$. The number of elements of $\mathcal {F}'$ contained in $T_1$ is at most $8\cdot 2^{-\ell }n$, so we indeed have $t\le 8\cdot 2^{-\ell }n$. $\square$

## Four cycle-free matrices

In this section, we prove Theorem 1.5. Most of this section is devoted to proving that four cycle-free matrices of average degree *d* have $\gamma _2$-norm at least $\Omega (\sqrt{d})$. From this, Theorem 1.5 follows after a bit of work.

We adapt certain graph terminology for Boolean matrices. By the average degree of a matrix, we mean the average degree of the bipartite graph whose bi-adjacency matrix is *M*. Let *M* be a matrix of average degree at least *d*. The first step is to find a submatrix of average degree $\Omega (d)$ where either every row contains $\Theta (d')$ one entries, or every column contains $\Theta (d')$ entries for some $d'=\Omega (d)$. Unfortunately, it is a well known result of graph theory [36] that it is not always possible to find a submatrix in which this is true for both the rows and columns simultaneously, which would also make our proof significantly simpler.

### Lemma 7.1

Let *M* be a Boolean matrix of average degree at least *d*. Then *M* contains a submatrix *N* of average degree $d'\ge d/3$ such that every row and column of *N* contains at least $d'/2$ one entries, and either $||N||_{{{\,\textrm{row}\,}}}^2\le 6d'$ or $||N||_{{{\,\textrm{col}\,}}}^2\le 6d'$.

### Proof

Let *G* be the bipartite graph, whose bi-adjacency matrix is *M*, and let *A* and *B* be the vertex classes of *G*. Our task is to show that *G* contains an induced subgraph $G'$ of average degree $d'\ge d/3$ such that $G'$ has minimum degree at least $d'/2$, and every degree in one of the parts is at most $6d'$.

Let $G_0$ be an induced subgraph of *G* of maximum average degree and let $d_0$ be the average degree of $G_0$, so that $d_0\ge d$. First, we note that $G_0$ has no vertex of degree less than $d_0/2$. Indeed, otherwise, if $v\in V(G_0)$ is such a vertex, then the average degree of $G_0-v$ (i.e., the graph we get by removing *v*) has average degree $2e(G_0-v)/(v(G_0)-1)>(2e(G_0)-d_0)/(v(G_0)-1)=d_0$.

Let $A_0\subset A,B_0\subset B$ be the vertex classes of $G_0$, and assume without loss of generality that $|A_0|\ge |B_0|$. Note that the number of edges of $G_0$ is $\frac{d_0}{2}(|A_0|+|B_0|)$. If we let $C\subset A_0$ be the set of vertices of degree more than $2d_0$, then $|C|\le |A_0|/2$. Indeed, otherwise, the number of edges of $G_0$ is at least $2d_0|C|>d_0|A_0|\ge \frac{d_0}{2}(|A_0|+|B_0|)$, a contradiction. Let $A_1=A_0\setminus C$, and let $G_1$ be the subgraph of $G_0$ induced on $A_1\cup B_0$. The number of edges of $G_1$ is at least $d_0|A_1|/2\ge d_0 (|A_1|+|B_0|)/6$, so the average degree of $G_1$ is at least $d_0/3$. Let $G'$ be an induced subgraph of $G_1$ of maximum average degree, and let $d'$ be the average degree of $G'$. Then $d'\ge d_0/3$, and every vertex of $G'$ has degree at least $d'/2\ge d_0/6\ge d/6$. Furthermore, if $A'\subset A_1$ and $B'\subset B_0$ are the vertex classes of $G'$, then every degree in $A'$ is at most $2d_0\le 6d'$. This finishes the proof. $\square$

Now the idea of the proof is as follows. After passing to a submatrix *N* which is close to regular from one side, say all columns have $\Theta (d')$ one entries, we use the fact that

$$\begin{aligned} \gamma _2(N)\ge \left| \left| N\circ \left( u v^T\right) \right| \right| _{{{\,\textrm{tr}\,}}} \end{aligned}$$

for any choice of unit vectors *u* and *v* (of the appropriate dimensions), see property (4) of the $\gamma _2$-norm. We choose *u* to be a vector whose entries are based on the degree distribution of the rows, and choose *v* to be the normalized all-ones vector. Letting $A=N\circ (u v^T)$, we then inspect the matrices $B=AA^T$ and $B^2$. With the help of the Cauchy interlacing theorem, we show that the singular values of *A* follow a certain distribution and thus find a lower bound for $||A||_{{{\,\textrm{tr}\,}}}$.

### Lemma 7.2

Let *M* be a four cycle-free Boolean matrix of average degree at least *d*. Then

$$\begin{aligned} \gamma _2(M)=\Omega \left( \sqrt{d}\right) . \end{aligned}$$

### Proof

Let *N* be a submatrix of *M* satisfying the outcome of Lemma 7.1. Then if we let $d_0$ be the average degree of *N*, we have $d_0\ge d/3$, every row and column of *N* contains at least $d_0/2$ one entries, and $||N||_{{{\,\textrm{row}\,}}}^2\le 6d_0$ or $||N||_{{{\,\textrm{col}\,}}}^2\le 6d_0$. Without loss of generality, we assume that $||N||_{{{\,\textrm{col}\,}}}^2\le 6d_0$. In what follows, we only work with the matrix *N*, and our goal is to prove that $\gamma _2(N)=\Omega (\sqrt{d_0})$, which will then imply that $\gamma _2(M)=\Omega (\sqrt{d})$. To simplify notation, we write *d* instead of $d_0$.

Let the size of *N* be $m\times n$, and recall that for any choice of $u\in \mathbb {R}^m$ and $v\in \mathbb {R}^n$ with $||u||=||v||=1$, we have

$$\begin{aligned} \gamma _2(N)\ge \left| \left| M\circ \left( uv^T\right) \right| \right| _{{{\,\textrm{tr}\,}}}. \end{aligned}$$

Let *f* be the number of one entries of *N*, and for $i=1,\dots ,m$, let $d_i$ be the number of one entries of row *i*. Note that $f=d_1+\dots +d_m$. Let $u\in \mathbb {R}^m$ be defined as $u(i)=\sqrt{d_i/f}$ for $i\in [m]$, and let $v\in \mathbb {R}^n$ be defined as $v(i)=1/\sqrt{n}$. Then we have $||u||=||v||=1$. If we let $A=N\circ (uv^T)$, then $\gamma _2(N)\ge ||A||_{{{\,\textrm{tr}\,}}}$, so it is enough to prove that $||A||_{{{\,\textrm{tr}\,}}}=\Omega (\sqrt{d})$. Note that if we let $\sigma _1\ge \dots \ge \sigma _m\ge 0$ be the singular values of *A* (possibly with zeros added to get exactly *m* of them), then $\sigma _1^2,\dots ,\sigma _m^2$ are the eigenvalues of $B=AA^T$.

Define the auxiliary graph *H* on [*m*], where for $i,i'\in [m]$, $i\ne i'$, we put an edge between *i* and $i'$ if there is some index $j\in [n]$ such that $N(i,j)=N(i',j)=1$. As *M* is four cycle-free, there is at most one such index *j* for every pair $(i,i')$. With this notation, we can write

$$B\left( i,i'\right) =\frac{1}{fn}{\left\{ \begin{array}{ll} d_i^2 &  \text{ if } i=i' \\ \sqrt{d_id_{i'}} &  \text{ if } i\sim i' \\ 0 &  \text{ otherwise. }\end{array}\right. }$$

For $t=1,\dots ,\lceil \log _3 n\rceil =:p$, let $I_t\subset [m]$ be the set of indices *i* such that $3^{t-1}< d_i\le 3^t$. Then $I_1,\dots ,I_p$ forms a partition of [*m*], and we note that $I_t$ is empty if $t\le \log _3 d-1$. Let $B_t=B[I_t\times I_t]$, then $B_t$ is a principal submatrix of *B*.

### Claim 7.3

At least $\Omega (|I_t|)$ eigenvalues of $B_t$ are at least $\Omega (3^{2t}/(fn))$.

### Proof

Let $s=|I_t|$, $D=3^{t-1}$, and let $\lambda _1\ge \dots \ge \lambda _s\ge 0$ be the eigenvalues of $B_t$. Then

3 $$\begin{aligned} \lambda _1+\dots +\lambda _s={{\,\textrm{tr}\,}}(B_t)=\frac{1}{fn}\sum _{i\in I_t}d_i^2\ge \frac{sD^2}{fn}, \end{aligned}$$

and

$$\begin{aligned} \lambda _1^2+\dots +\lambda _s^2=\left| \left| B_t\right| \right| _F^2. \end{aligned}$$

Here,

$$\begin{aligned} \left| \left| B_t\right| \right| _F^2= &   \sum _{i,i'\in I_t}B\left( i,i'\right) ^2=\frac{1}{(fn)^2}\left[ \sum _{i\in I_t}d_i^4+\sum _{i\sim i',i,i'\in I_t}d_id_{i'} \right] \\\le &   \frac{1}{(fn)^2}\left[ 81sD^{4}+18e\left( H\left[ I_t\right] \right) D^{2}\right] ,\end{aligned}$$

where $e(H[I_t])$ denotes the number of edges of the subgraph of *H* induced on the vertex set $I_t$. Let *G* be the bipartite graph, whose bi-adjacency matrix is $N[I_t\times [n]]$. Then $e(H[I_t])$ is the number of pairs $\{i,i'\}\in I_t^{(2)}$ such that *i* and $i'$ has a common neighbour in *G*. As every column of *N* has at most 6*d* one entries, every vertex in [*n*] has degree at most 6*d* in *G*. Thus, for each $i\in I_t$, there are at most $6dd_i\le 18dD$ vertices $i'\in I_t$ which have a common neighbour with *i*. Therefore, $e(H[I_t])\le 12dDs\le 36D^2s$, where we used in the last inequality that $s=0$ unless $t\ge \log _3 d-1$. In conclusion, we proved that

$$\begin{aligned} \lambda _1^2+\dots +\lambda _s^2\le \frac{1000sD^4}{(fn)^2}. \end{aligned}$$

Let $C=2000$, and let $r\le s$ be the largest index such that $\lambda _r\ge \frac{CD^2}{fn}$. By the previous inequality, we have $r\le \frac{1000s}{C^2}$. But then by the inequality between the arithmetic and square mean, we have

$$\begin{aligned} \lambda _1+\dots +\lambda _r\le r^{1/2}\left( \lambda _1^2+\dots +\lambda _r^2\right) ^{1/2}\le r^{1/2}\left( \frac{1000sD^4}{(fn)^2}\right) ^{1/2}\le \frac{sD^2}{2fn}. \end{aligned}$$

Hence, comparing this with (3), we deduce that

$$\begin{aligned} \lambda _{r+1}+\dots +\lambda _{s}\ge \frac{sD^2}{2fn}. \end{aligned}$$

As $\frac{CD^2}{fn}\ge \lambda _{r+1}\ge \dots \ge \lambda _s$, this is only possible if at least $\frac{s}{4C}$ among $\lambda _{r+1},\dots ,\lambda _s$ is at least $\frac{D^2}{4fn}$. This finishes the proof. $\square$

As $B_t$ is a principal submatrix of *B*, its eigenvalues interlace the eigenvalues of *B*, see Lemma 3.2. Therefore, the previous claim implies that at least $c|I_t|$ eigenvalues of *B* are at least $c3^{2t}/(fn)$ for some absolute constant $c>0$, and thus at least $c|I_t|$ singular values of *A* are at least $c3^t/\sqrt{fn}$.

We are almost done. Note that

$$\begin{aligned} \sum _{t=1}^p 3^{t-1}|I_t|\le f=\sum _{i=1}^md_i\le \sum _{t=1}^p 3^{t}|I_t|. \end{aligned}$$

In order to bound $||A||_{{{\,\textrm{tr}\,}}}=\sigma _1+\dots +\sigma _m$, we observe that for every *t*, if

$$\begin{aligned} |I_t|\ge \max \left\{ |I_{t+1}|,\dots ,|I_p|\right\} =:z_t, \end{aligned}$$

then

$$\begin{aligned} \sum _{i=z_t+1}^{|I_t|}\sigma _i\ge \frac{c3^t}{\sqrt{fn}}\cdot \left( c\left| I_t\right| -c\left| I_{t+1}\right| -\dots -c\left| I_p\right| \right) . \end{aligned}$$

Hence,

$$\begin{aligned} ||A||_{{{\,\textrm{tr}\,}}}&=\sum _{i=1}^m \sigma _i\ge \sum _{t=1}^p \frac{c3^t}{\sqrt{fn}}\cdot \left( c\left| I_t\right| -c\left| I_{t+1}\right| -c\left| I_{t+2}\right| -\dots -c\left| I_{p}\right| \right) \\&\ge \frac{c^2}{\sqrt{fn}}\sum _{t=1}^p \left| I_t\right| \left( 3^t-3^{t-1}-\dots -3-1\right) \ge \frac{c^2}{\sqrt{fn}}\sum _{t=1}^p\frac{3^t}{2} |I_t|\ge \frac{c^2\sqrt{f}}{2\sqrt{n}}. \end{aligned}$$

Here, in the first inequality, we use that if $|I_t|< z_t$, then the contribution of the *t*-th term in the sum is anyway negative. Finally, recall that every column of *N* contains at least *d*/2 one entries, so $f\ge dn/2$. Therefore, $||A||_{{{\,\textrm{tr}\,}}}\ge \frac{c^2}{12}\sqrt{d}$, finishing the proof. $\square$

### Proof of Theorem 1.5

First, we prove that $\gamma _2(M)\le 2\sqrt{d}$. Let *G* be the bipartite graph, whose bi-adjacency matrix is *M*, and let *A* and *B* be the vertex classes of *G*, where *A* corresponds to the rows of *M*, and *B* corresponds to the columns. Then there is an ordering < of $A\cup B$ such that every vertex $v\in A\cup B$ has at most *d* neighbours larger than *v* in this ordering. Let $G_1$ be the subgraph of *G* on vertex set $A\cup B$ in which we keep those edges *ab* with $a\in A$ and $b\in B$, where $a<b$, and let $G_2$ be the graph containing the rest of the edges. Let $M_i$ be the bi-adjacency matrix of $G_i$ for $i=1,2$, then $M=M_1+M_2$, every row of $M_1$ has at most *d* one entries, and every column of $G_2$ has at most *d* one entries. In particular, $\gamma (M)\le \gamma (M_1)+\gamma (M_2)\le 2\sqrt{d}$.

Second, we prove that $\gamma _2(M)=\Omega (\sqrt{d})$. If we let *N* be a submatrix of *M* of minimum degree *d*, then the average degree of *N* is at least *d*. Hence, Lemma 7.2 implies the desired lower bound by noting that $\gamma _2(M)\ge \gamma _2(N)$. $\square$

Finally, we prove Theorem 1.6. The key graph theoretical result tying Theorem 1.5 and Theorem 5.2 together is the following lemma of [32].

### Lemma 7.4

(Hunter, Milojević, Sudakov, Tomon [32]) For every *k* there exists $c=c(k)>0$ such that the following holds. Let *G* be a bipartite graph of average degree *d*, which contains no four cycle-free induced subgraph with average degree at least *k*. Then *G* contains a subgraph on at most *d* vertices with at least $cd^2$ edges.

### Proof of Theorem 1.6

Let *G* be the bipartite graph, whose bi-adjacency matrix is *M*, and let $d\ge Ct$ be the average degree of *G*, where $C=C(\gamma )$ is specified later. By Theorem 1.5, there exists $c_0>0$ such that *G* contains no four-cycle free induced subgraph with average degree at least $k=c_0\gamma ^2$. If we let $c=c(k)$ be the constant guaranteed by Lemma 7.4, then *G* contains a subgraph *H* with at most *d* vertices, and at least $cd^2$ edges. Then *H* corresponds to a $d_1\times d_2$ submatrix *N* of *M* with at least $cd^2$ one entries, where $d_1+d_2\le d$. We want to find a large all-ones submatrix of *N*. We may assume that $d_1=d_2=d$ by adding all-zero rows and columns to *N*. If we let $N'=J-N$, then $\gamma _2(N')\le \gamma +1$, and $N'$ has at least $cd^2$ zero entries. In other words, $p(N')\le 1-c$. By applying Lemma 4.4 to $N'$ with $\varepsilon =c$ and $\delta =1/2$, we can find an $\alpha d\times \alpha d$ sized submatrix $N''$ of *N* such that $\alpha =2^{-O(\gamma /c^2)}$ and $p(N'')<1/2$. If we let $n'=\alpha d$, then Theorem 5.2 implies that $N''$ contains a $t'\times t'$ all-zeros submatrix for some $t'=n'2^{-O(\gamma ^3)}\ge \lambda d$, where $\lambda =\lambda (\gamma )>0$ only depends on $\gamma$. In conclusion, choosing $C=1/\lambda$, we have $t'\ge t$, and thus *M* contains a $t\times t$ all-ones submatrix. $\square$

## Graphs of bounded smallest eigenvalue

In this section, we prove Theorem 2.4. First, we present a technical lemma, which we use to construct a large class of graphs that are forbidden in graphs of bounded smallest eigenvalue.

### Lemma 8.1

Let *H* be a graph satisfying the following. The vertex set of *H* can be partitioned into three *k* element sets *A*, *B*, *C* such that there are no edges between *A* and *B*, but the bipartite subgraph between $A\cup B$ and *C* is complete (and we make no assumption about the edges in *A*, *B* or *C*). Then the smallest eigenvalue of *H* is at most $-k/3$.

### Proof

Let *A* be the adjacency matrix of *H*, and let $x\in \mathbb {R}^{V(H)}$ be the vector defined as $x(v)=1/\sqrt{3k}$ if $v\in A\cup B$, and $x(v)=-1/\sqrt{3k}$ if $v\in C$. Observe that $||x||=1$. Let $-\lambda$ be the smallest eigenvalue of *A*, then

$$\begin{aligned} {-}\lambda&\le x^TAx{=} \frac{2}{3k}(e(G[A]){+}e(G[B]){+}e(G[C]){+}e(G[A,B]){-}e(G[A\cup B,C])){=}\\&{=}\frac{2}{3k}\left( e(G[A])+e(G[B])+e(G[C])-2k^2\right) \\&\le \frac{2}{3k}\left( \frac{3k^2}{2}-2k^2\right) =-\frac{k}{3}. \end{aligned}$$

$$\square $$

Next, we show that the adjacency matrix of a graph with bounded smallest eigenvalue has bounded $\gamma _2$-norm as well.

### Lemma 8.2

Let $\lambda >0$, let *G* be a graph and let *A* be the adjacency matrix of *G*. If the smallest eigenvalue of *G* is at least $-\lambda$, then $\gamma _2(A)\le 2\lambda$.

### Proof

If *M* is an $n \times n$ symmetric positive semidefinite matrix, it is not hard to show that $\gamma _2(M) = \max _{1\le i\le n}{M_{i,i}}$, which was first proved by Schur [54] in 1911. Therefore, as $A+\lambda I$ is positive semidefinite, we have $\gamma _2(A + \lambda I) = \lambda$. By the subadditivity of the norm,

$$\begin{aligned} \gamma _2(A) \le \gamma _2(A + \lambda I) + \gamma _2(-\lambda I) = 2 \lambda . \end{aligned}$$

$$\square $$

### Proof of Theorem 2.4

We may assume that *d* is sufficiently large with respect to $\lambda$, otherwise the statement is trivial. Let *A* be the adjacency matrix of *G*, then $\gamma _2(A)\le 2\lambda$. Hence, by Theorem 1.6, *A* contains a $t\times t$ all-ones submatrix for some $t=\Omega _{\lambda }(d)$. But then this corresponds to a complete bipartite subgraph in *G* with vertex classes *X*, *Y* such that $|X|=|Y|=t$. We assume that *d* is sufficiently large such that $t\ge 3\lambda +1$ is satisfied.

### Claim 8.3

If $k=\lceil 3\lambda +1\rceil$, then *X* cannot contain two disjoint *k* element sets with no edges between them.

### Proof

Assume to the contrary that there exist $A,B\subset X$ with no edges between them, and $|A|=|B|=k$. If *C* is any *k* element subset of *Y*, then $A\cup B\cup C$ spans a subgraph *H* of *G* fitting the description of Lemma 8.1. Thus the smallest eigenvalue of *H* is at most $-k/3< -\lambda$. But then as *H* is an induced subgraph of *G*, the Cauchy interlacing theorem (Lemma 3.2) implies that the smallest eigenvalue of *G* is less than $-\lambda$, contradiction. $\square$

Let $G'$ the complement of *G*[*X*], and let *D* be the average degree of $G'$. The adjacency matrix of $G'$ has $\gamma _2$-norm at most $2\lambda +2$. Hence, applying Theorem 1.6 to $G'$, we deduce that $G'$ contains a complete bipartite graph with vertex classes of size $u=\Omega _{\lambda }(D)$. But this gives two *u*-element sets in *G*[*X*] with no edges between them. Therefore, by the previous claim, $u\le 3\lambda +1$, and in particular $D=O_{\lambda }(1)$. Applying Turán’s theorem to *G*[*X*], we conclude that *G*[*X*] contains a complete subgraph of size at least

$$\begin{aligned} \frac{|X|}{D+1}=\Omega _{\lambda }(d), \end{aligned}$$

finishing the proof. $\square$

## Inverse theorem for MaxCut

In this section, we prove Theorem 1.7. Let us introduce some notation. Given a graph *G*, the *MaxCut* of *G* is the maximum number of edges in a *cut* of *G*, where a cut is a partition of *V*(*G*) into two sets, with all edges having exactly one endpoint in both parts. We denote by ${{\,\textrm{mc}\,}}(G)$ the MaxCut of *G*, and furthermore, if *G* has *m* edges, we define the *surplus* of *G* as

$$\begin{aligned} {{\,\textrm{sp}\,}}(G)={{\,\textrm{mc}\,}}(G)-m/2. \end{aligned}$$

In case the vertices of *G* are partitioned randomly, the expected number of edges in the resulting cut is *m*/2. This implies that ${{\,\textrm{sp}\,}}(G)\ge 0$. The following is equivalent to Theorem 1.7.

### Theorem 9.1

Let *G* be a graph with *m* edges such that ${{\,\textrm{sp}\,}}(G)\le \alpha \sqrt{m}$. Then *G* contains a clique of size at least $2^{-O(\alpha ^9)}\sqrt{m}$.

We prepare the proof of this theorem by collecting a few simple properties of the surplus.

### Lemma 9.2

Let *G* be a graph, and let $V_1,\dots ,V_k$ be disjoint subsets of *V*(*G*). Then

$$\begin{aligned} {{\,\textrm{sp}\,}}(G)\ge \sum _{i=1}^k{{\,\textrm{sp}\,}}\left( G\left[ V_i\right] \right) . \end{aligned}$$

### Proof

For $i=1,\dots ,k$, let $(A_i,B_i)$ be a partition of $V_i$ witnessing the MaxCut of $G[V_i]$. Define the partition (*X*, *Y*) of *V*(*G*) as follows: for every $i\in [k]$, let either $A_i\subset X$ and $B_i\subset Y$ or $A_i\subset Y$ and $B_i\subset X$ independently with probability 1/2. Also, add every vertex not contained in $\bigcup _{i=1}^k V_i$ to either *X* or *Y* with probability 1/2. Observe that if *f* is an edge of *G* not contained in $G[V_i]$ for any $i\in [k]$, then *f* is cut with probability 1/2. Otherwise, if $f\subset V_i$, then *f* is cut if and only if *f* is cut by $(A_i,B_i)$. Hence, the expected size of the cut (*X*, *Y*) is exactly $|E(G)|/2+\sum _{i=1}^k {{\,\textrm{sp}\,}}(G[V_i]))$. $\square$

### Lemma 9.3

(Erdős, Gyárfás, Kohayakawa [16]) Let *G* be a graph on *n* vertices with no isolated vertices. Then ${{\,\textrm{sp}\,}}(G)\ge n/6$.

The main technical lemma underpinning the proof of Theorem 9.1 is the following result showing that the surplus can be lower bounded by the energy of *G*. The *energy* of *G* is the trace-norm of the adjacency matrix of *G*, or equivalently, the sum of absolute values of the adjacency matrix, and it is denoted by $\mathcal {E}(G)$. A similar, but weaker result was proved recently by Räty and Tomon [53], who showed that if *G* has *n* vertices, then ${{\,\textrm{sp}\,}}(G)=\Omega (\mathcal {E}(G)/\log n)$. Here, we prove that the $1/\log n$ factor can be removed. Our proof follows the semidefinite programming approach of [53]. However, to remove the $1/\log n$ factor, which comes from an application of the Graph Grothendieck inequality [3], we instead combine this idea with the MaxCut approximation approach of Goemans and Williamson [30].

### Lemma 9.4

Let *G* be a graph. Then ${{\,\textrm{sp}\,}}(G)=\Omega (\mathcal {E}(G))$.

### Proof

We recall the following identity: if $x,y\in \mathbb {R}^n$, then $\langle xx^T,yy^T\rangle =\langle x,y\rangle ^2$.

We may assume that *G* contains no isolated vertices, as the removal of such vertices does not change the energy or the surplus. Let $\{1,\dots ,n\}$ be the vertex set of *G*, and let *A* be its adjacency matrix. Let $\lambda _1\ge \dots \ge \lambda _n$ be the eigenvalues of *A* with a corresponding orthonormal basis of eigenvectors $v_1,\dots ,v_n$. Define the symmetric positive semidefinite matrix

$$\begin{aligned} M=\sum _{i: \lambda _i<0} v_iv_i^T. \end{aligned}$$

We make a number of observations about *M*. First of all,

$$\begin{aligned} \langle A,M\rangle {=}\left\langle \sum _{i{=}1}^n \lambda _i v_iv_i^T,\sum _{i:\lambda _i{<}0}v_iv_i^T\right\rangle {=}\sum _{i{=}1}^n\sum _{j:\lambda _j<0}\lambda _i\langle v_i,v_j\rangle ^2=\sum _{i:\lambda _i<0}\lambda _i{=}-\mathcal {E}(G)/2. \end{aligned}$$

In the last equality, we used that well known fact that $0=\text{ trace }(A)=\sum _{i=1}^{n}\lambda _i$. Moreover,

$$\begin{aligned} ||M||_F^2=\langle M,M\rangle =\sum _{i:\lambda _i<0}1\le n. \end{aligned}$$

Lastly, $M_{i,i}\le 1$ for $i\in [n]$, as $I-M=\sum _{i:\lambda _i\ge 0}v_iv_i^T$ is positive semidefinite. Let $M'$ be the $n\times n$ matrix such that $M'_{i,i}=1$ for $i\in [n]$ and $M'_{i,j}=M_{i,j}$ if $i,j\in [n],i\ne j$. Then $M'$ is positive semidefinite, $\langle A,M'\rangle =\langle A,M\rangle$, and $||M'||_F^2\le 2n$. In particular, there exist unit vectors $x_1,\dots ,x_n\in \mathbb {R}^n$ such that $M'_{i,j}=\langle x_i,x_j\rangle$ for every $i,j\in [n]$.

Using the vectors $x_1,\dots ,x_n$, we describe a probability distribution on the cuts of *G* as follows. Let $\textbf{H}$ be a random linear hyperplane in $\mathbb {R}^n$, then $\textbf{H}$ cuts the *n*-dimensional space into two parts. We define the partition (*X*, *Y*) of *V*(*G*) such that *X* contains the vertices corresponding to the vectors $x_1,\dots ,x_n$ in one of the parts, while *Y* is the set of vertices corresponding to the vectors in the other part. Formally, we take $\textbf{u}$ randomly on the unit sphere $\mathbb {S}^{n-1}$ from the uniform distribution, and set

$$\begin{aligned} X=\{i\in [n]: \langle x_i,\textbf{u}\rangle \ge 0\} \text{ and } Y=\{i\in [n]:\langle x_i,\textbf{u}\rangle <0\}. \end{aligned}$$

Given an edge $f=\{a,b\}$, consider the probability that *f* is cut by (*X*, *Y*). This probability can be calculated easily as follows: let $\alpha =\arccos (\langle x_a,x_b\rangle )$ be the angle between $x_a$ and $x_b$, then

$$\begin{aligned} \mathbb {P}(f \text{ is } \text{ cut } \text{ by } (X,Y))=\frac{\alpha }{\pi }. \end{aligned}$$

By the Taylor expansion of the function $\arccos (.)$, we can write

$$\begin{aligned} \arccos (t)=\frac{\pi }{2}-t-O\left( t^2\right) . \end{aligned}$$

The error term can be improved to $O(t^3)$, however, quadratic decay already suffices for our purposes. From this, we get

$$\begin{aligned} \mathbb {P}(f \text{ is } \text{ cut } \text{ by } (X,Y))=\frac{1}{2}-\frac{\langle x_a,x_b\rangle }{\pi }-O\left( \left\langle x_a,x_b\right\rangle ^2\right) . \end{aligned}$$

Let $\textbf{T}$ be the number of edges in the cut (*X*, *Y*), then

$$\begin{aligned} \mathbb {E}(\textbf{T})&{=}\sum _{\{a,b\}\in E(G)} \mathbb {P}(\{a,b\} \text{ is } \text{ cut } \text{ by } (X,Y)){=}\sum _{\{a,b\}\in E(G)}\frac{1}{2}{-}\frac{\langle x_a,x_b\rangle }{\pi }{-}O\left( \left\langle x_a,x_b\right\rangle ^2\right) \\&=\frac{e(G)}{2}{-}\frac{\left\langle A,M'\right\rangle }{\pi }{-}O\left( \left| \left| M'\right| \right| _F^2\right) {=}\frac{e(G)}{2}+\frac{\mathcal {E}(G)}{2\pi }-O(n). \end{aligned}$$

Therefore, as there exists a cut (*X*, *Y*) with at least $\mathbb {E}(\textbf{T})$ edges, we arrive to the inequality

$$\begin{aligned} {{\,\textrm{sp}\,}}(G)\ge \frac{\mathcal {E}(G)}{2\pi }-O(n). \end{aligned}$$

By Lemma 9.3, we also have ${{\,\textrm{sp}\,}}(G)=\Omega (n)$, which combined with the previous inequality gives the desired bound ${{\,\textrm{sp}\,}}(G)=\Omega (\mathcal {E}(G))$. $\square$

### Proof of Theorem 9.1

Let *n* be the number of vertices of *G*. Without loss of generality, we may assume that *G* does not contain isolated vertices. Then, by Lemma 9.3, we have ${{\,\textrm{sp}\,}}(G)\ge n/6$. Hence,

$$\begin{aligned} \sqrt{m}\le n\le 6\alpha \sqrt{m}, \end{aligned}$$

where the first inequality is true for every graph, and the second inequality is due to our assumption ${{\,\textrm{sp}\,}}(G)\le \alpha \sqrt{m}$. By Lemma 9.4, there exists a constant $c_0>0$ such that $c_0{{\,\textrm{sp}\,}}(G)\ge \mathcal {E}(G)$, so

$$\begin{aligned} \mathcal {E}(G)\le c_0\alpha \sqrt{m}\le c_0\alpha n. \end{aligned}$$

Let *A* be the adjacency matrix of *G*, then $\mathcal {E}(G)=||A||_{{{\,\textrm{tr}\,}}}\le c_0\alpha n$. Hence, by Lemma 5.1, for every $\varepsilon >0$, there exists a $n'\times n'$ submatrix *B* of *A* such that $\gamma _2(B)\le c_0\alpha /\varepsilon$ and $n'\ge (1-\varepsilon )n$. Here, as the removal of $\varepsilon n$ rows and columns can destroy at most $2 \varepsilon n^2$ one entries, we have

$$\begin{aligned} |B|\ge |A|-2\varepsilon n^2\ge |A|-72\alpha ^2\varepsilon m=m\left( 2-72\alpha ^2\varepsilon \right) . \end{aligned}$$

Choosing $\varepsilon =1/72\alpha ^2$, we get a submatrix *B* such that

$$\begin{aligned} |B|\ge m\ge \frac{1}{36\alpha ^2}\left( n'\right) ^2 \end{aligned}$$

and $\gamma :=\gamma _2(B)\le 72c_0\alpha ^3$. Next, we want to find a large submatrix $B'$ such that $p(B')\ge 1/2$. In order to do this, we apply Lemma 4.4 to $J-B$. As $p(J-B)\le 1-\frac{1}{36\alpha ^2}$, we can find such a submatrix $B'$ of size $n''\times n''$, where $n''=2^{-O(\gamma \alpha ^4)}n'=2^{-O(\alpha ^7)}\sqrt{m}$. Now we can apply Theorem 5.2 to $J-B'$ to conclude that $B'$, and thus *A*, contains $t\times t$ sized all-ones submatrix with $t=2^{-O(\gamma ^3)}n''=2^{-O(\alpha ^9)}\sqrt{m}$.

This all-ones submatrix corresponds to a complete bipartite subgraph of *G*, let *X* and *Y* be its vertex classes, and note that $|X|=|Y|=t$. Let $H=G[X\cup Y]$. By Lemma 9.2, ${{\,\textrm{sp}\,}}(G)\ge {{\,\textrm{sp}\,}}(H)$, so ${{\,\textrm{sp}\,}}(H)\le \alpha \sqrt{m}$. On the other hand, by considering the cut (*X*, *Y*), we have ${{\,\textrm{mc}\,}}(H)\ge t^2$, so

$$\begin{aligned} {{\,\textrm{sp}\,}}(H)&={{\,\textrm{mc}\,}}(H)-\frac{|E(H)|}{2}\ge t^2-\frac{t^2+e(G[X])+e(G[Y])}{2} \\&= \frac{t^2}{2}-\frac{e(G[X])}{2}-\frac{e(G[Y])}{2}. \end{aligned}$$

Write $$e(G[X])=\left( {\begin{array}{c}t\\ 2\end{array}}\right) -x$$ and $$e(G[Y])=\left( {\begin{array}{c}t\\ 2\end{array}}\right) -y$$. Then using the inequality ${{\,\textrm{sp}\,}}(H)\le \alpha \sqrt{m}$, the previous inequality becomes

$$\begin{aligned} \alpha \sqrt{m}\ge \frac{t+x+y}{2}\ge \frac{x}{2}. \end{aligned}$$

In particular, we showed that the complement of *G*[*X*] has at most $2\alpha \sqrt{m}$ edges. By Turán’s theorem (Theorem 3.1), then *G*[*X*] contains a clique of size at least

$$\begin{aligned} \frac{t^2}{4\alpha \sqrt{m}+t}=2^{-O(\alpha ^9)}\sqrt{m}. \end{aligned}$$

This finishes the proof. $\square$

## Data Availability

The manuscript has no associated data.
